# Novel chromosomal insertions of IS*Ecp1*-*bla*_CTX-M-15_ and diverse antimicrobial resistance genes in Zambian clinical isolates of *Enterobacter cloacae* and *Escherichia coli*

**DOI:** 10.1186/s13756-021-00941-8

**Published:** 2021-05-10

**Authors:** Misheck Shawa, Yoshikazu Furuta, Gillan Mulenga, Maron Mubanga, Evans Mulenga, Tuvshinzaya Zorigt, Christone Kaile, Manyando Simbotwe, Atmika Paudel, Bernard Hang’ombe, Hideaki Higashi

**Affiliations:** 1 Division of Infection and Immunity, Research Center for Zoonosis Control, Hokkaido University, Sapporo, Japan; 2 Department of Para-Clinical Studies, School of Veterinary Medicine, University of Zambia, Lusaka, Zambia; 3 Department of Disease Control, School of Veterinary Medicine, University of Zambia, Lusaka, Zambia; 4 Department of Pathology and Microbiology, University Teaching Hospital, Lusaka, Zambia

**Keywords:** IS*Ecp1*, *bla*_CTX-M_, Chromosomal insertion, Extended spectrum β-lactamase, Zambia, AMR, *Enterobacter cloacae*, *Escherichia coli*

## Abstract

**Background:**

The epidemiology of extended-spectrum β-lactamases (ESBLs) has undergone dramatic changes, with CTX-M-type enzymes prevailing over other types. *bla*_CTX-M_ genes, encoding CTX-M-type ESBLs, are usually found on plasmids, but chromosomal location is becoming common. Given that *bla*_CTX-M_-harboring strains often exhibit multidrug resistance (MDR), it is important to investigate the association between chromosomally integrated *bla*_CTX-M_ and the presence of additional antimicrobial resistance (AMR) genes, and to identify other relevant genetic elements.

**Methods:**

A total of 46 clinical isolates of cefotaxime-resistant *Enterobacteriaceae* (1 *Enterobacter cloacae*, 9 *Klebsiella pneumoniae*, and 36 *Escherichia coli*) from Zambia were subjected to whole-genome sequencing (WGS) using MiSeq and MinION. By reconstructing nearly complete genomes, *bla*_CTX-M_ genes were categorized as either chromosomal or plasmid-borne.

**Results:**

WGS-based genotyping identified 58 AMR genes, including four *bla*_CTX-M_ alleles (i.e., *bla*_CTX-M-14_, *bla*_CTX-M-15_, *bla*_CTX-M-27_, and *bla*_CTX-M-55_). Hierarchical clustering using selected phenotypic and genotypic characteristics suggested clonal dissemination of *bla*_CTX-M_ genes. Out of 45 *bla*_CTX-M_ gene-carrying strains, 7 harbored the gene in their chromosome. In one *E. cloacae* and three *E. coli* strains, chromosomal *bla*_CTX-M-15_ was located on insertions longer than 10 kb. These insertions were bounded by IS*Ecp1* at one end, exhibited a high degree of nucleotide sequence homology with previously reported plasmids, and carried multiple AMR genes that corresponded with phenotypic AMR profiles.

**Conclusion:**

Our study revealed the co-occurrence of IS*Ecp1*-*bla*_CTX-M-15_ and multiple AMR genes on chromosomal insertions in *E. cloacae* and *E. coli*, suggesting that ISE*cp1* may be responsible for the transposition of diverse AMR genes from plasmids to chromosomes. Stable retention of such insertions in chromosomes may facilitate the successful propagation of MDR clones among these *Enterobacteriaceae* species.

**Supplementary Information:**

The online version contains supplementary material available at 10.1186/s13756-021-00941-8.

## Background

Extended-spectrum β-lactamases (ESBLs) are bacterial enzymes capable of degrading most β-lactam antibiotics, rendering them therapeutically useless. Many environmental [1] and animal [2] reservoirs of ESBLs have been reported, however, the heavy use of antimicrobials in hospitals remains the main driver of ESBL-mediated antimicrobial resistance (AMR) [3, 4]. The problem of ESBLs is emerging globally [5, 6] and represents one of the most dire current public health threats in both community and hospital settings [7–9]. ESBLs have been identified and described in both industrialized and resource-constrained countries. For example, a high ESBL burden has been observed in France and China, where local hospitals reported prevalence figures of 17.7% [10] and 68.2% [11], respectively. Likewise, studies among hospital patients in Africa show ESBL occurrences as high as 62.3% in Mali [12], 70% in Burkina Faso [13], and 84% in Cote d’Ivoire [14]. In reports from Zambia, ESBL-associated multidrug resistance (MDR) was observed in all 45 *Klebsiella pneumoniae* isolates obtained from neonates [15] and in all 15 diarrhoeagenic *Escherichia coli* isolates from under-five children [16] at the University Teaching Hospital (UTH).

Over 200 ESBLs have been documented [17], of which the majority are derivatives of TEM, SHV, or CTX-M. While TEM and SHV are common ESBL variants, the CTX-M family has recently emerged to become the preponderant type worldwide [18]. This epidemiologic transition has been paralleled by the success and rapid dissemination of the pandemic clone *E. coli* O25b:H4-ST131, which is frequently associated with *bla*_CTX-M-15_ [19]. Additionally, the efficient spread of *bla*_CTX-M_ genes has also been facilitated by plasmids, integrons, and insertion sequences (IS) (e.g., IS*Ecp1*, IS*1*, IS*5*, and IS*26*) [20]. *bla*_CTX-M_ genes are usually associated with MDR, since they primarily reside on plasmids carrying other AMR genes. Considering that plasmids often incur a fitness cost in the absence of antibiotic selection pressure [21], it is possible to combat *bla*_CTX-M_-associated MDR through the prudent use of antimicrobials. However, a series of studies have reported the occurrence of *bla*_CTX-M_ genes on the chromosomes of various *Enterobacteriaceae* [22–27], although their placement with other AMR genes was not examined. With a growing number of studies reporting the chromosomal location of *bla*_CTX-M_, it is of considerable interest to explore possible links between chromosomally-located *bla*_CTX-M_ and MDR.

In this study, using phenotypic and genotypic characterization of clinical *Enterobacteriaceae* isolates from Zambia, we identified the probable clonal spread of *bla*_CTX-M_ among selected strains. Furthermore, by reconstructing and analyzing nearly complete genome sequences, we identified chromosomally-integrated plasmid segments co-harboring IS*Ecp1*-*bla*_CTX-M-15_ and diverse AMR genes in *Enterobacter cloacae* and *E. coli*. Most of the strains harboring these insertions exhibited MDR phenotypes corresponding with the AMR genes in the insertions. Based on these findings, we speculate that IS*Ecp1*-mediated transposition may be responsible for the spread of MDR determinants among *Enterobacteriaceae*. Furthermore, stable maintenance of these chromosomal insertions may enhance the dissemination of multiple MDR *Enterobacteriaceae* species, with potentially detrimental effects on patient outcomes.

## Methods

### Strain selection

Between June and October 2018, 46 non-repetitive cefotaxime resistant clinical *Enterobacteriaceae* strains were obtained from the UTH, Zambia. These strains were previously isolated from blood (*n* = 1), cerebrospinal fluid (CSF) (*n* = 2), high vaginal swab (HVS) (*n* = 1), pus (*n* = 3), sputum (*n* = 4), stool (*n* = 30), and urine (*n* = 5) during routine clinical investigations. Cefotaxime resistance was confirmed by plating each strain on LB agar supplemented with 1 μg/ml cefotaxime (Sigma-Aldrich, USA). In addition, the minimum inhibitory concentration (MIC) of cefotaxime against each strain was determined by broth microdilution.

### MIC determination

Antimicrobial susceptibility profiles of strains toward 10 different antimicrobials were determined by broth microdilution using breakpoints specified in Additional file 1: Table S1. Briefly, overnight cultures were diluted 10^4^-fold, added to twofold serial dilutions of antibiotics in a 96-well plate in triplicate, then incubated at 37 °C for 18 h with shaking at 1600 rpm. Next, optical densities at 595 nm (OD_595_) were measured using the Multiskan FC Microplate Photometer (Thermo Scientific, USA), with OD_595_ values of at least 0.1 indicating positive bacterial growth. The MIC was defined as the minimum antibiotic concentration producing an OD_595_ value of less than 0.1. Two laboratory strains, *E. coli* MG1655 and *E. coli* 10-β (NEB, USA), were used for quality control.

### Growth rate determination

Bacterial cultures prepared in plain LB were monitored for growth by measuring OD_600_ in real-time over a period of 16 h. To this end, overnight cultures were diluted 10^3^-fold and added to a 96-well plate in duplicate, then subjected to OD monitoring using the Varioskan LUX Multimode Microplate Reader (Thermo Scientific, USA) at 37 °C, while shaking at 600 rpm. Growth rates were estimated from the slopes obtained by fitting parametric models to the resulting data using the R package grofit version 1.1.1 [28].

### Whole genome sequencing

Genomic DNA was extracted from overnight cultures prepared in LB supplemented with 1 μg/ml cefotaxime using a QIAamp PowerFecal DNA Kit (QIAGEN). Libraries were prepared using NexteraXT (Illumina) and sequenced using MiSeq (Illumina) to obtain 300 bp paired-reads. The same genomic DNA samples were also subjected to long-read sequencing by MinION (Oxford Nanopore Technologies) using a Rapid Barcoding Kit (SQK-RBK004) and an R9.5 flowcell (FLO-MIN107). Short-read trimming and adapter sequence removal were performed using Trim Galore version 0.4.2 with options of "–paired –nextera" (https://github.com/FelixKrueger/TrimGalore). Basecalling of long reads was performed using Guppy Basecalling Software version 3.4.5 and the reads were assembled using Canu version 1.8 [29] with the "corOutCoverage = 1000 genomeSize = 6 m" option applied. Redundant repeats at terminal ends of contigs were identified using Gepard version 1.40 [30] and manually trimmed down to one copy. Trimmed contigs were then subjected to base-error correction with Illumina reads using Pilon version 1.23 [31]. Contigs longer than 2 Mb were classified as chromosomal while circular contigs of less than 500 kb and containing plasmid replicons were classified as plasmids. To categorize the rest of the contigs as either chromosomal or plasmid-based in origin, a local database was generated for each strain using known chromosomal and plasmid segments. Unclassified contigs were then subjected to BLASTn searches against the local database and sequences matching known chromosome or plasmid regions with at least 70% identity were regarded as redundant and removed from the data pool. Sequences exhibiting hits with less than 70% identity were retained and subjected to NCBI BLASTn searches against the nt database. A sequence was regarded as chromosomal if at least 70% of the top 10 hits were chromosomes, and as plasmid-based if at least 70% of the top 10 hits were plasmids.

### Phylogenetic analysis

To elucidate the evolutionary relationships among strains, whole genome-based phylogenetic trees were constructed using Parsnp version 1.2 [32] and imported into MEGA version 7.0 [33] for visualization and editing. Multilocus sequence typing (MLST) was performed in silico by uploading raw Illumina reads to a public MLST server (www.cbs.dtu.dk/services/MLST) [34].

### Detection of plasmid replicons, strain serotypes, and AMR genes

Plasmid replicons and O:H serotypes were determined by interrogating contigs with PlasmidFinder [35] and EcOH [36] databases, respectively, using ABRicate software version 0.8.10 (https://github.com/tseemann/abricate) with options –mincov 90 and –minid 90 specified. To identify AMR genes, the AMRFinderPlus tool [37] was used with option -i 0.7 engaged.

### Determination of clustering patterns among strains

To unravel the mechanisms of *bla*_CTX-M_ spread at the UTH, strains were compared in terms of AMR phenotype, AMR genes, and plasmid replicons by exploiting clustering patterns and correlations using the R package ComplexHeatmap [38].

### Sequence alignment and determination of chromosomal insertion locations of ***bla***_CTX-M_

Strains carrying chromosomal *bla*_CTX-M_ were annotated using DFAST version 1.2.4 [39] and aligned to reference sequences using Mauve [40]. Zam_UTH_18, Zam_UTH_26, and Zam_UTH_41 were aligned against the reference strain *E. coli* MG1655 (GenBank accession no. NC_000913.2), while Zam_UTH_44 was aligned against *E. cloacae* ATCC 13047 (GenBank accession no. NC_014121.1). Zam_UTH_42 and Zam_UTH_47 were aligned against *E. coli* ST648 (GenBank accession no. CP008697.1), while Zam_UTH_43 was aligned against another strain (Zam_UTH_08) in our data. Comparison, visualization, and exploration of the data were performed using genoPlotR [41].

### PCR and sanger sequencing

To validate and further characterize chromosomal insertions, junctions between chromosomes and plasmids were amplified by PCR using primers shown in Supplementary Additional file 1: Table S2. Where possible, primers external to the insertion were used and amplicon size was compared to that obtained from a control strain. Also, strains for which the exact *bla*_CTX-M_ allele was unidentifiable due to low Illumina read number were subjected to PCR using primers shown in Additional file 1: Table S3, followed by Sanger sequencing. Briefly, PCR was performed using KOD One Master Mix (TOYOBO, Japan) and the products were purified using a MinElute PCR Purification Kit (QIAGEN). Sequencing PCR was performed using a BigDye Terminator v3.1 Cycle Sequencing Kit (Applied Biosystems, USA), followed by Sanger sequencing using a 3130 Genetic Analyzer (Applied Biosystems, USA). Raw sequence data was assembled using SnapGene software (Insightful Science, available at snapgene.com) and the obtained consensus sequences analyzed by the AMRFinderPlus tool [37] with option -i 0.7.

## Results

### MLST revealed high genetic diversity among strains

This study examined 46 cefotaxime-resistant *Enterobacteriaceae* strains isolated from various clinical samples among patients at the UTH, Zambia (Table 1). To elucidate the genetic variability and relationships among these strains, WGS was used to reconstruct nearly complete genome sequences. In silico MLST from the WGS data identified the strains as *E. cloacae* (1/46, 2.2%), *K. pneumoniae* (9/46, 19.6%), and *E. coli* (36/46, 78.3%) (Table 1).

**Table 1:** Description of 46 strains used in this study

Strain ID	Source	Species	CTX MIC^a^	Growth rate	Patient	
					Age^b^	Gender
Zam_UTH_01	Stool	*E. coli*	16	0.126	54	M
Zam_UTH_02	Stool	*K. pneumoniae*	≥ 512	0.147	21	M
Zam_UTH_03	Urine	*E. coli*	≥ 512	0.206	25	F
Zam_UTH_04	Stool	*K. pneumoniae*	≥ 512	0.071	28	M
Zam_UTH_05	Stool	*K. pneumoniae*	≥ 512	0.255	52	M
Zam_UTH_06	Urine	*E. coli*	≥ 512	0.166	8	M
Zam_UTH_07	Stool	*K. pneumoniae*	≥ 512	0.101	1	F
Zam_UTH_08	Stool	*E. coli*	≥ 512	0.117	46	F
Zam_UTH_09	Stool	*K. pneumoniae*	128	0.512	36	M
Zam_UTH_10	Stool	*K. pneumoniae*	≥ 512	0.231	54	F
Zam_UTH_11	Stool	*E. coli*	≥ 512	0.149	65	M
Zam_UTH_12	HVS	*E. coli*	256	0.213	23	F
Zam_UTH_13	Stool	*E. coli*	256	0.140	43	F
Zam_UTH_15	Urine	*E. coli*	256	0.120	40	M
Zam_UTH_17	Stool	*E. coli*	256	0.168	3	M
Zam_UTH_18	Pus	*E. coli*	256	0.225	25	F
Zam_UTH_20	Stool	*E. coli*	≥ 512	0.132	92	F
Zam_UTH_21	Stool	*E. coli*	256	0.112	11	M
Zam_UTH_22	Stool	*E. coli*	256	0.195	13	F
Zam_UTH_23	Pus	*E. coli*	≥ 512	0.188	5	M
Zam_UTH_24	Stool	*E. coli*	128	0.185	8	M
Zam_UTH_25	Stool	*E. coli*	128	0.167	73	M
Zam_UTH_26	Stool	*E. coli*	256	0.189	1	F
Zam_UTH_27	Stool	*E. coli*	256	0.118	36	M
Zam_UTH_28	Stool	*E. coli*	64	0.226	36	M
Zam_UTH_29	Urine	*E. coli*	128	0.379	44	F
Zam_UTH_30	Stool	*K. pneumoniae*	≥ 512	0.060	52	F
Zam_UTH_31	Stool	*E. coli*	≥ 512	0.237	25	M
Zam_UTH_32	Stool	*E. coli*	128	0.297	6	F
Zam_UTH_33	Stool	*E. coli*	≥ 512	0.154	70	M
Zam_UTH_34	Stool	*E. coli*	128	0.220	11	F
Zam_UTH_36	Stool	*E. coli*	128	0.285	73	M
Zam_UTH_37	Stool	*K. pneumoniae*	≥ 512	0.254	1	M
Zam_UTH_38	Sputum	*E. coli*	256	0.223	32	M
Zam_UTH_39	Pus	*E. coli*	≥ 512	0.131	64	F
Zam_UTH_40	CSF	*K. pneumoniae*	≥ 512	0.107	N/A	F
Zam_UTH_41	Stool	*E. coli*	≥ 512	0.136	N/A	F
Zam_UTH_42	Pus	*E. coli*	≥ 512	0.145	N/A	M
Zam_UTH_43	Urine	*E. coli*	256	0.230	N/A	F
Zam_UTH_44	Stool	*E. cloacae*	128	0.163	1	M
Zam_UTH_45	Sputum	*E. coli*	256	0.176	32	M
Zam_UTH_46	Sputum	*E. coli*	128	0.160	1	M
Zam_UTH_47	Stool	*E. coli*	≥ 512	0.172	1	M
Zam_UTH_48	Sputum	*E. coli*	128	0.298	27	F
Zam_UTH_50	CSF	*E. coli*	128	0.222	7	M
Zam_UTH_51	Blood	*E. coli*	128	0.221	2	F

^a^CTX MIC is expressed in μg/ml

^b^N/A = not available

**Fig. 1:**
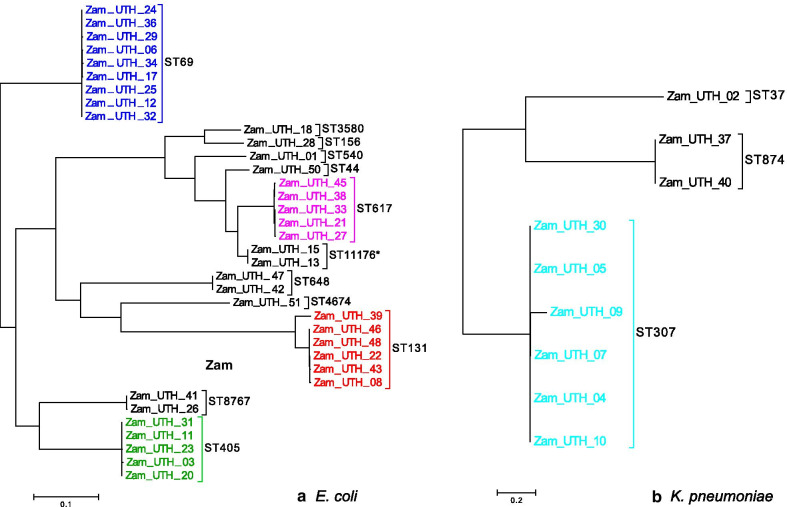
Phylogenetic analysis. Whole genome-based phylogenetic trees for 36 *E. coli* and 9 K*. pneumoniae* strains*.* **a** *E. coli*: a total of 12 STs were identified, including one novel type (marked with *). Four STs constituted 25/36 (69.4%) of *E. coli* strains, the most common being ST69 (9 strains), followed by ST131 (6 strains). **b** *K. pneumoniae*: a total of 3 STs were identified, with ST307 alone representing 6/9 (66.7%) strains

Although *E. coli* were represented by 12 sequence types (STs), 25/36 (69.4%) strains belonged to 4 STs (i.e., ST69, ST131, ST617, and ST405) (Fig. 1a), suggesting the expansion of a few eminent clones. ST131 is known to rapidly spread due to its diverse virulence and AMR mechanisms [42], but its prevalence in this study, at 6/36 (16.7%), was second to that of ST69. ST69 predominated, with a prevalence of 9/36 (25.0%), highlighting its important contribution to the burden of ESBLs in the local hospital. Of the 12 *E. coli* STs identified in this study, one was novel and has since been designated ST11176. Among the *K. pneumoniae* isolates, which were classified into 3 STs, the most prevalent was ST307, representing 6/9 (66.7%) strains (Fig. 1b).

### Chromosomal ***bla***_CTX-M_ genes were identified by in silico analysis

**Fig. 2:**
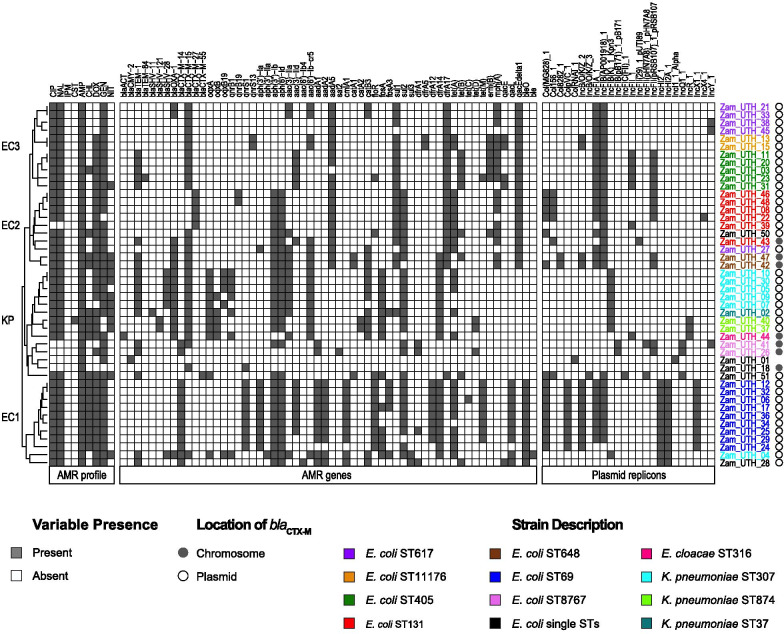
AMR phenotypes, AMR genes and plasmid replicons. All but one strain displayed resistance to at least three antimicrobial classes. There was no phenotypic or genotypic resistance to imipenem, however, one strain (Zam_UTH_40) exhibited phenotypic resistance to colistin. A total of 12 AMR gene classes were identified. Within the β-lactamase gene class, the *bla*_CTX-M_ family showed the most diversity, with *bla*_CTX-M-15_ being the most common variant. Most *bla*_CTX-M_ genes were located on plasmids, however, 7/45 (15.6%) strains harbored the genes on the chromosome. A total of 24 plasmid replicons were detected, with the most prevalent being IncFIB(AP001918)_1, which was present in 30/46 (65.2%) strains. Hierarchical clustering showed aggregation of strains of the same ST. Cefotaxime (CTX) is not shown here since all strains were selected using CTX. AMP; ampicillin. CHL; chloramphenicol. CIP; ciprofloxacin. CST; colistin. DOX; doxycycline. GEN; gentamicin. IPM; imipenem. NAL; nalidixic acid. NIT; nitrofurantoin

To identify AMR gene patterns among the strains, in silico detection was performed using AMR gene sequence databases. A total of 58 AMR genes belonging to 12 classes were identified (Additional file 1: Table S4). Among the β-lactamase genes, the *bla*_CTX-M_ family predominated, with 45/46 (97.8%) strains carrying at least one of the following alleles: *bla*_CTX-M-14_, *bla*_CTX-M-15_, *bla*_CTX-M-27_, and *bla*_CTX-M-55_ (Fig. 2). These *bla*_CTX-M_ genes were located on chromosomes in 7 strains (7/45, 15.6%; one *E. cloacae* and six *E. coli*); one of these strains harbored an extra *bla*_CTX-M_ gene on a plasmid, and the remaining 38 strains (38/45, 84.4%) harbored *bla*_CTX-M_ genes exclusively on plasmids. In agreement with previous studies [43], *bla*_CTX-M-15_ was the most prevalent ESBL gene (28/45, 62.2%). Despite documentation of a close association between *bla*_CTX-M-15_ gene and *E. coli* ST131 [44], only one *E. coli* ST131 (1/6, 16.7%) possessed *bla*_CTX-M-15_ (Fig. 2), with the remaining five *E. coli* ST131 strains possessing plasmid-borne *bla*_CTX-M-27_. In silico serotyping of all six *E. coli* ST131 strains revealed that none of them belonged to the pandemic clone O25b:H4-ST131, with the 5 *bla*_CTX-M-27_-carrying strains belonging to Onovel31:H4 and the *bla*_CTX-M-15_-carrying strain belonging to O107:H5.

To determine antimicrobial susceptibility profiles, MICs of 10 antimicrobials were measured against each strain. Forty-five out of 46 (97.8%) strains displayed MDR phenotypes, defined as a lack of susceptibility to at least one antimicrobial from at least three antimicrobial classes [45]. The highest non-susceptibility rate was to ampicillin (46/46, 100%), followed by gentamicin (43/46, 93.5%), ciprofloxacin (41/46, 89.1%), and nalidixic acid (41/46, 89.1%). While no carbapenem resistance was detected, one *K. pneumoniae* strain (1/46, 2.2%) displayed low-level resistance (MIC = 4 μg/ml) to the drug of last resort, colistin (Fig. 2). In almost all cases the probability of phenotypic resistance was high (positive predictive value ≥ 80%) given a positive genotypic result (Additional file 1: Table S5). While previous studies have shown a significant correlation between resistance range and fitness cost [46], we did not find any correlation between the number of AMR genes and bacterial growth rate (Additional file 1: Fig. S1A).

### ***E. coli*** ST69 strains possess an IncH plasmid harboring ***bla***_CTX-M-14_

To characterize the plasmid content of the strains, WGS data were compared against the PlasmidFinder database [35]. In total, 24 plasmid replicons were detected, the most common being IncFIB(AP001918)_1 (30/46, 65.2%), IncFIA_1 (27/46, 58.7%), Col(MG828)_1 (16/46, 34.8%), and IncB/O/K/Z_2 (13/46, 28.3%) (Fig. 2). Most *E. coli* ST69 strains (8/9, 88.0%) in this study carried an IncF plasmid possessing *dfrA12* and *sul2* genes, encoding resistance to trimethoprim and sulfamethoxazole, respectively. This was expected since previous studies have associated *E. coli* ST69 with IncF plasmids encoding trimethoprim-sulfamethoxazole (SXT) resistance [47]. However, all the *E. coli* ST69 strains in this study also carried two additional MDR plasmids, including a 225 kb IncH plasmid which is not commonly found in this ST [47, 48]. This IncH plasmid contained *bla*_CTX-M-14_ and shared over 80% homology with an IncH plasmid in a *K. pneumoniae* ST37 strain (Zam_UTH_04) from our data pool (Additional file 1: Fig. S2A), indicating that these plasmids could share a common origin. The two plasmids exhibited similar genetic contexts around *bla*_CTX-M-14_ gene, however, both plasmids contained several insertions that carried various AMR genes as well as genes related to fitness traits (Additional file 1: Fig. S2B). For instance, the IncH plasmid from *E. coli* ST69 possessed the *mucAB* operon which is associated with resistance to DNA-damaging agents such as ultraviolet radiation [49]. Furthermore, this plasmid also harbored the *mer* operon that is involved in resistance to organomercury compounds [50].

Analysis of the other three dominant *E. coli* STs (i.e., ST131, ST617, and ST405) showed a close association between IncF plasmids and *bla*_CTX-M_ genes, as previously reported [51]. However, comparison of IncF plasmids from strains of different STs revealed low nucleotide sequence homology (Additional file 1: Fig. S2C), ruling out horizontal gene transfer as a mode of *bla*_CTX-M_ gene propagation.

### The spread of ***bla***_CTX-M_ genes is driven by clonal expansion

To establish the mode of *bla*_CTX-M_ propagation among strains, hierarchical clustering was conducted using AMR phenotypes, AMR genes, and plasmid replicons as variables. The results indicated that strains can be grouped into 4 main clusters, which we designated as EC1, EC2, EC3, and KP (Fig. 2). The largest cluster, EC1, was predominated by *E. coli* ST69 and was characterized by the presence of *bla*_CTX-M-14_ and *IncHI*. In addition, EC1 contained more AMR genes than what was observed in clusters EC2, EC3, and KP (*P* < 0.01) (Additional file 1: Fig. S3). Cluster EC2 was closely related to EC3, with both clusters containing *qacEdelta1*, *mph(A)*, and *aad5* genes that were absent in clusters EC1 and KP. Lastly, cluster KP, formed by *K. pneumoniae* and *E. cloacae*, was defined by the presence of four plasmid-mediated quinolone resistance (PMQR) genes, *oqxA*, *oqxB*, *oqxB19*, and *qnrB1*. Overall, the analysis showed the aggregation of strains belonging to the same ST. Taken together with our MLST findings, these results suggest the possible spread of *bla*_CTX-M_ by clonal expansion rather than horizontal gene transfer.

### Large chromosomal insertions co-harbor ***bla***_CTX-M-15_ and other AMR genes

**Fig. 3:**
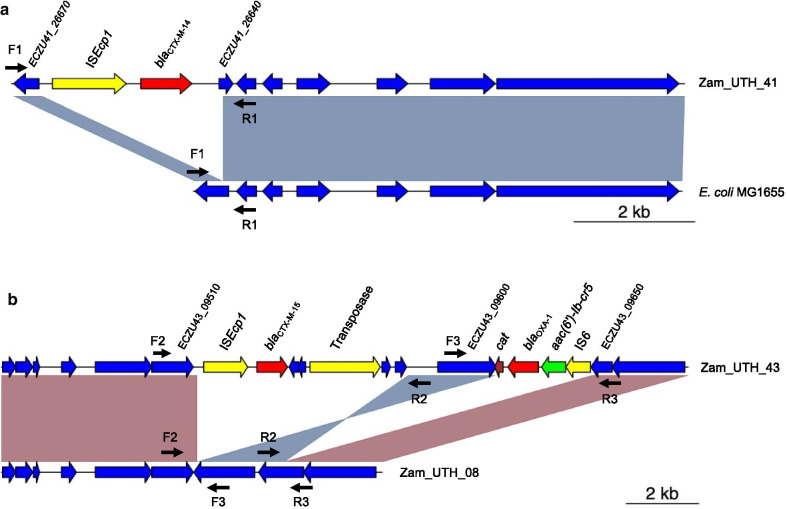
*bla*_CTX-M_ genes present on short chromosomal insertions in *E. coli*. **a** Zam_UTH_41. This *E. coli* ST8767 strain carried *bla*_CTX-M-14_ on a 3,095 bp chromosomal insertion with IS*Ecp1* located 249 bp upstream of *bla*_CTX-M-14_. Zam_UTH_26 (not shown) also had a similar genetic context for its insertion. **b** Zam_UTH_43. This *E. coli* ST131 O107:H5 strain harbored *bla*_CTX-M-15_ on a 6,036 bp chromosomal insertion, with IS*Ecp1* located 255 bp upstream of *bla*_CTX-M-15_. About 2.5 kb downstream of this insertion was another insertion that harbored genes conferring resistance to chloramphenicol (*cat*), β-lactams (*bla*_OXA-1_), and aminoglycosides/quinolones (*aac(6′)-Ib-cr5*). F1, F2, F3, R1, R2, R3; primers used for confirmation of insertions

**Fig. 4:**
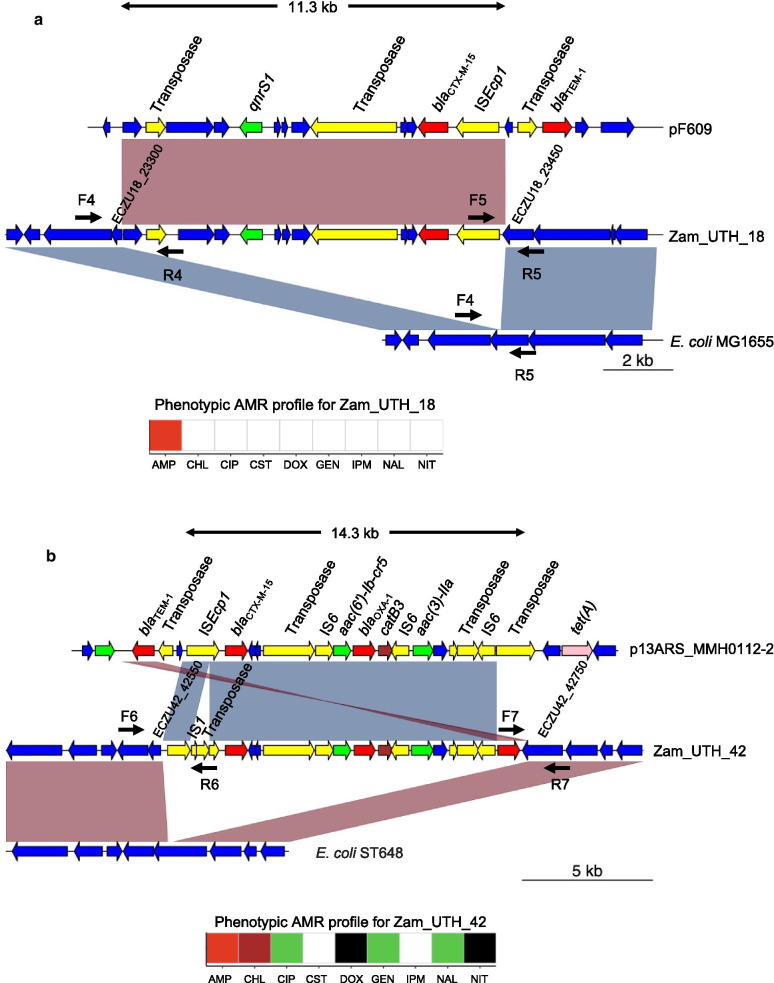
*bla*_CTX-M_ genes present on large chromosomal insertions in *E. coli*. **a** Zam_UTH_18. This *E. coli* ST3580 strain possessed *bla*_CTX-M-15_ on an 11,383 bp chromosomal insertion, which was very similar to plasmid pF609 (GenBank accession no. MK965545.1). *bla*_CTX-M-15_ was closely associated with IS*Ecp1*, which was located 255 bp upstream. The insertion also harbored the quinolone resistance gene *qnrS1*, located 4639 bp downstream of *bla*_CTX-M-15_. The phenotypic AMR profile of this strain showed resistance to ampicillin and susceptibility to quinolones. **b** Zam_UTH_42. This *E. coli* ST648 strain harbored *bla*_CTX-M-15_ on a 14,328 bp chromosomal insertion that was very similar to plasmid p13ARS_MMH0112-2 (GenBank accession no. LR697123.1). This insertion carried genes associated with resistance to aminoglycosides (*aac(3)-IIa*)*,* aminoglycosides/quinolones (*aac(6′)-Ib-cr5*), β-lactams (*bla*_OXA-1_, *bla*_TEM-1_), and chloramphenicol (*catB3*). IS*Ecp1* was located 255 bp upstream of *bla*_CTX-M-15_, however, unlike in other strains, IS*Ecp1* in this strain was truncated by IS*1* and transposase. The phenotypic AMR profile of this strain was consistent with the AMR genotype for the insertion. Zam_UTH_47 (not shown) also had a similar genetic context for its insertion. F4, F5, F6, F7, R4, R5, R6, R7; primers used for confirmation of insertions. White; susceptible. Black; resistance phenotype in the absence of corresponding AMR gene. Red; β-lactam resistance. Brown; chloramphenicol resistance. Green; aminoglycoside and/or quinolone resistance

**Fig. 5:**
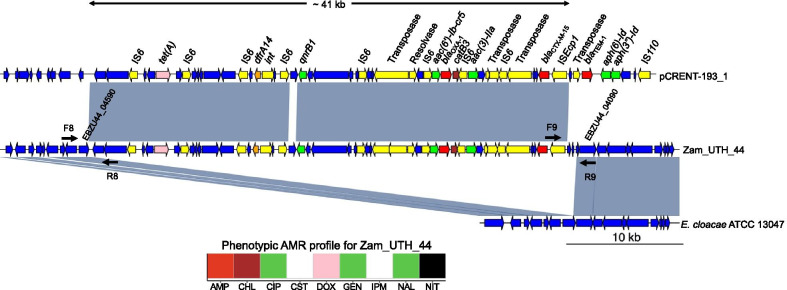
*bla*_CTX-M_ gene on a large chromosomal insertion in *E. cloacae*. Zam_UTH_44. This *E. cloacae* ST316 strain carried *bla*_CTX-M-15_ on a ~ 41 kb chromosomal insertion that exhibited nucleotide sequence homology with plasmid pCRENT-193_1 (GenBank accession no. CP024813.1). IS*Ecp1* was located 255 bp upstream of *bla*_CTX-M-15_. The insertion also included diverse AMR genes encoding resistance to six antimicrobial classes, namely; aminoglycosides (*aac(3)-IIa*)*,* quinolones (*qnrB1*), aminoglycosides/quinolones (*aac(6′)-Ib-cr5*)*,* β-lactams (*bla*_OXA-1_), trimethoprim (*dfrA14*), chloramphenicol (*catB3*), and tetracyclines (*tet(A)*). The phenotypic AMR profile of this strain was consistent with the AMR genotype of the insertion. F8, F9, R8, R9; primers used for confirmation of insertions. White; susceptible. Black; resistance phenotype in the absence of corresponding AMR gene. Red; β-lactam resistance. Brown; chloramphenicol resistance. Green; aminoglycoside and/or quinolone resistance. Pink; tetracycline resistance

To determine the genetic contexts of chromosomally-located *bla*_CTX-M_ in 7 strains (one *E. cloacae* and six *E. coli*), chromosomal insertions were identified by alignment against reference sequences using Mauve [40]. In all cases, the insertions were bounded by IS*Ecp1* at one end (Figs. 3, 4, 5), implying that this element is responsible for mobilization. However, some insertions also harbored various IS elements (e.g., IS*1*, IS*6*) and transposons, suggesting that other sophisticated mechanisms may play a role. The inserted segments, which were confirmed by PCR (Additional file 1: Fig. S4), were genetically distinct, with sizes ranging from ~ 3 kb to ~ 41 kb. Furthermore, these insertions showed high nucleotide sequence similarity to plasmids available in the NCBI GenBank, implying the transposition from plasmid to chromosome. In one *E. cloacae* and three *E. coli* strains, *bla*_CTX-M_-carrying insertions were larger than 10 kb and harbored other types of AMR genes. As expected, strains possessing these MDR insertions showed resistance to multiple antibiotics of clinical importance (Figs. 4, 5).

Chromosomal insertions were bracketed by 5-bp direct repeats in all six *E. coli* strains, which is typical of IS*Ecp1*-mediated transposition. However, IS*Ecp1* was truncated by IS*1* and transposase in two strains (Zam_UTH_42 and Zam_UTH_47) (Fig. 4b), suggesting that the interrupted IS*Ecp1* may no longer be functional, and that mobilization of *bla*_CTX-M-15_ may have been induced by IS*1* and/or transposase. In all strains, the relative position of the downstream *bla*_CTX-M_ gene was constant for each allele (i.e., *bla*_CTX-M-14_ or *bla*_CTX-M-15_), indicating possible allele-specific ancestral origins. For instance, in Zam_UTH_26 and Zam_UTH_41 (both *E. coli* ST8767), *bla*_CTX-M-14_ was located 249 bp downstream of IS*Ecp1* on a 3,095 bp insertion. Similarly, in 5 strains, *bla*_CTX-M-15_ was observed 255 bp downstream of IS*Ecp1* on insertions of varying lengths. More specifically, Zam_UTH_43 (*E. coli* ST131) (Fig. 3b) and Zam_UTH_18 (*E. coli* ST3580) (Fig. 4a) carried *bla*_CTX-M-15_ on 6,036 bp and 11,383 bp insertions, respectively, while Zam_UTH_42 and Zam_UTH_47 (both *E. coli* ST648) (Fig. 4b) carried *bla*_CTX-M-15_ on a 14,328 bp insertion. Furthermore, Zam_UTH_44 (*E. cloacae* ST316) (Fig. 5) carried *bla*_CTX-M-15_ on a chromosomal insertion longer than 41 kb.

Of the 7 strains carrying chromosomal *bla*_CTX-M_, 4 strains (i.e., Zam_UTH_18, Zam_UTH_42, Zam_UTH_44, and Zam_UTH_47) harbored the gene on insertions longer than 10 kb that exhibited high nucleotide sequence similarity to plasmids available in the NCBI GenBank (Figs. 3, 4, 5). Notably, the insertion in Zam_UTH_18 also appeared in the chromosome sequences of *Salmonella enterica* (GenBank accession no. CP045038) and two *E. coli* ST38 strains (GenBank accession no. CP010116 and CP018976). Furthermore, on the 4 insertions larger than 10 kb, we found additional AMR genes downstream of *bla*_CTX-M-15_. Zam_UTH_18 was found to possess the *qnrS1* gene, which is associated with quinolone resistance, while Zam_UTH_42 and Zam_UTH_47 had genes associated with resistance to aminoglycosides (*aac(3)-IIa*)*,* aminoglycosides/quinolones (*aac(6′)-Ib-cr5*), β-lactams (*bla*_OXA-1_, *bla*_TEM-1_), and chloramphenicol (*catB3*). Similarly, Zam_UTH_44 possessed 7 genes known to confer resistance to aminoglycosides (*aac(3)-IIa*)*,* quinolones (*qnrB1*), aminoglycosides/quinolones (*aac(6′)-Ib-cr5*)*,* β-lactams (*bla*_OXA-1_), trimethoprim (*dfrA14*), chloramphenicol (*catB3*), and tetracyclines (*tet(A)*).

Phenotypic AMR profiles of the strains carrying chromosomal *bla*_CTX-M_ were in agreement with the presence of AMR genes on insertions except for Zam_UTH_18 which showed quinolone susceptibility despite possessing *qnrS1*. Additionally, these strains had cefotaxime MICs that were at least 64-fold higher than the clinical breakpoint of 2 μg/ml published by the Clinical and Laboratory Standards Institute [52]. Though Zam_UTH_26 was closely related to Zam_UTH_41 (Fig. 1a), the latter had a higher cefotaxime MIC (≥ twofold) possibly due to the presence of plasmid-borne *bla*_CTX-M-15_, in addition to *bla*_CTX-M-14_ on the chromosome. This difference was also reflected by the lower growth rate (μ = 0.136) of Zam_UTH_41 compared to Zam_UTH_26 (μ = 0.189), suggesting a fitness cost imposed by the extra *bla*_CTX-M-15_-carrying plasmid. Likewise, Zam_UTH_18, which lacked plasmids, grew at a rate higher than the 75^th^ percentile of the estimated *E. coli* growth rates (Additional file 1: Fig. S1B; Additional file 2: Fig. S5).

## Discussion

In this study, we sought to investigate the phenotypic and genotypic characteristics of clinical *Enterobacteriaceae* strains from the UTH, Zambia. Our results suggest that the dissemination of *bla*_CTX-M_ has been mediated mainly by clonal expansion. While the majority of strains possessed *bla*_CTX-M_ genes on plasmids, 7 strains harbored the genes on chromosomes. Data obtained in previous studies indicate that the chromosomal incorporation of *bla*_CTX-M_ is usually mediated by IS*Ecp1*; however, the co-occurrence of IS*Ecp1*-*bla*_CTX-M_ and additional AMR genes has never been reported in *E. cloacae* or *E. coli*. Despite approaches enabling examination of the immediate flanking regions of chromosomal *bla*_CTX-M_, limitations of Southern hybridization and WGS using short reads make it difficult to identify and resolve large chromosomal insertions. By reconstructing nearly complete genomes, our analysis of the genetic context of chromosomally-located *bla*_CTX-M_ was significantly improved compared to previous analyses. In one *E. cloacae* and three *E. coli* strains, we found *bla*_CTX-M-15_ in large chromosomal insertions whose nucleotide sequences significantly resembled plasmids available in the NCBI GenBank. We also found that these insertions were bounded by IS*Ecp1* at one end and possessed additional AMR genes. Phenotypic AMR profiles of these strains showed near-perfect agreement with the observed chromosomal MDR genotypes. These findings suggest that IS*Ecp1* contributes to the dissemination of *bla*_CTX-M-15_-associated MDR determinants among various *Enterobacteriaceae* species.

Phylogenetic analysis highlighted the predominance of four *E. coli* STs (ST69, ST131, ST617, and ST405) and one *K. pneumoniae* ST (ST307), suggesting the clonal dissemination of a few particular strains. Fitting with this hypothesis, strain comparisons based on AMR phenotypes, AMR genes, and plasmid replicons generally showed clustering of strains according to ST. Most *E. coli* ST131 strains belong to the O25b:H4 pandemic clone which frequently harbors *bla*_CTX-M-15_ [42, 53]. However, the six *E. coli* ST131 strains examined in this study belonged to either Onovel31:H4 or O107:H5. Newer variants of *E. coli* ST131 have recently emerged [54], including Onovel31:H4 [55] detected in this study. To our knowledge, this is the first report of *E. coli* ST131 with serotype O107:H5.

The most abundant *E. coli* ST in this study, ST69, belonged to a clade that possessed more AMR genes compared to other clades (Additional file 1: Fig. S3). Generally, ST69 strains lack ESBL genes [56], are associated with community acquired infections [57], and carry IncF plasmids encoding SXT resistance [47]. Interestingly, the ST69 strains in this study carried *bla*_CTX-M-14_ on an IncH plasmid and showed the highest prevalence among the examined hospital isolates. This rare combination of IncH plasmids and *E. coli* ST69 was also reported in an Egyptian strain isolated from raw milk cheese [58], although the IncH plasmid (GenBank accession no. CP023143) in this strain was substantially different in AMR gene content. The observed predominance of ST69 in our study may be a consequence of selection pressure arising from the use of SXT for prophylaxis against *Pneumocystis jirovecii* (previously *carinii*) pneumonia (PCP). In Zambia, HIV infected or exposed patients at risk of developing PCP receive SXT prophylaxis for several months, or even years, until they are no longer at risk [59]. While SXT prophylaxis is highly beneficial, this practice has been associated with the development of SXT resistance in various bacterial species, including *E. coli* [60–62]. We speculate that the high proportion of ST69 observed here was a result of SXT-mediated selection, followed by acquisition of the *bla*_CTX-M-14_-containing IncH plasmid. The resulting ESBL phenotype, coupled with survival mechanisms such as error-prone mutagenesis mediated by *mucAB* operon, probably fostered the emergence of ST69 as a successful hospital clone.

The phenotypic and genotypic resistance observed in this study may be attributed to poor local antimicrobial stewardship. In Zambia, policies promoting the judicious use of antimicrobials are hardly adhered to and most drugs can be accessed without a prescription [63]. Moreover, a study conducted at the UTH reported a 53.7% antibiotic prescription rate [64], clearly surpassing the 30% rate recommended by the WHO [65]. Most serious bacterial infections among UTH inpatients are treated with third-generation cephalosporins (e.g., cefotaxime) [66], while quinolones (e.g., ciprofloxacin) are commonly used for outpatient infections [64]. Our results show that nearly 90% of cefotaxime-resistant strains were also resistant to quinolones. Meanwhile, carbapenem use at UTH is strictly monitored and prescription usually depends on robust laboratory evidence. Encouragingly, we did not detect carbapenem resistance either phenotypically or genotypically. However, it is noteworthy that carbapenem resistance has continued to emerge globally, even in Africa [67], resulting in the gradual re-introduction of colistin as a drug of last resort [68]. Despite this transition in treatment by many countries, there is no current report of the clinical use of colistin in Zambia. Nonetheless, we found borderline phenotypic colistin resistance in a *K. pneumoniae* strain (Zam_UTH_40) isolated from CSF. While this finding is of clinical significance, further studies are needed to verify this observation.

Systematic characterization of the diverse genetic environments of chromosomally-located *bla*_CTX-M_ revealed a close association with IS*Ecp1*, as previously reported [69]. Intriguingly, one *E. cloacae* and three *E. coli* strains carried both IS*Ecp1*-*bla*_CTX-M-15_ and other AMR genes on large chromosomal insertions that significantly resembled publicly available plasmid sequences. While this work was in progress, Goswami et al. described an *E. coli* strain possessing *bla*_CTX-M-15_ and four other AMR genes on a chromosomal insertion similar to a plasmid region, however, in contrast to our work, the mobilizing unit was IS*26* [47]. More recently, in South Korea, Yoon et al. observed the co-occurrence of IS*Ecp1*-*bla*_CTX-M-15_ and other AMR genes on chromosomal insertions in *K. pneumoniae* [70]. In contrast, none of the *K. pneumoniae* strains in our study harbored chromosomal *bla*_CTX-M_ genes, probably due to geographic variations between Zambia and South Korea. Here we report, for the first time, chromosomal integration of IS*Ecp1*-*bla*_CTX-M-15_-associated MDR elements in *E. cloacae* and *E. coli*. Since the identified MDR insertions were all bounded by IS*Ecp1* at one end, it is likely that this IS element was responsible for mobilization. However, the presence of other mobile elements, as well as the interruption of IS*Ecp1* in 2 strains, also potentially suggests the occurrence of other complex genetic events. Phenotypic susceptibilities confirmed that chromosomally-located MDR determinants were capable of conferring AMR to multiple drugs. For instance, Zam_UTH_44, which carried exclusively chromosomal AMR genes, displayed resistance to β-lactams, quinolones, gentamicin and chloramphenicol. However, Zam_UTH_18 was susceptible to quinolones despite harboring chromosomal *qnrS1*. We did not investigate *qnrS1* expression, however, this observed contradiction could be due to the fact that PMQR determinants (such as *qnrS1*) are associated with only small reductions in quinolone susceptibility [71]. Nevertheless, such small changes are usually sufficient to cause treatment failure in patients, and thus some researchers advocate for the revision of quinolone breakpoints [72].

While the benefits of chromosomal integration of IS*Ecp1*-*bla*_CTX-M-15_-associated MDR elements are not fully understood, it may help accelerate the spread of MDR determinants as an intermediary reservoir. As previously observed, plasmids belonging to the same incompatibility group are unable to stably coexist in the same bacterial host cell line [73]. Interplasmid gene transfer would thus be optimized by transient chromosomal incorporation. Furthermore, chromosomal integration of IS*Ecp1*-*bla*_CTX-M-15_-associated MDR elements warrants stable dissemination of resistant strains regardless of the presence or absence of antibiotic selection pressure [70]. Since bacteria are likely to lose plasmids when the cost of maintaining them outweighs their benefits [74, 75], chromosomal integration and the stable maintenance of crucial AMR genes seem to be a viable safeguard for survival and continued AMR spread. This hypothesis is supported by our observation that Zam_UTH_18 did not carry any plasmids but possessed a chromosomally-incorporated 11 kb segment bearing *bla*_CTX-M-15_ and *qnrS1*. The high growth rate exhibited by this strain may suggest a fitness advantage conferred by its plasmid-free status. We speculate that Zam_UTH_18 may have possessed a plasmid that was lost after chromosomal integration of the *bla*_CTX-M-15_-carrying segment. To verify this observation, more studies need to be conducted on growth and competitive performance using a reference strain harboring the *bla*_CTX-M-15_-containing segment on a plasmid.

**Table 2:** Accession numbers for reads and assemblies

Strain ID	MiSeq biosample	MinION biosample	Assembly accession numbers
Zam_UTH_01	SAMD00243947	SAMD00243947	BNEX01000001–BNEX01000005
Zam_UTH_02	SAMD00243948	SAMD00243948	BNEY01000001–BNEY01000008
Zam_UTH_03	SAMD00243949	SAMD00243949	BNEZ01000001–BNEZ01000005
Zam_UTH_04	SAMD00243950	SAMD00243950	BNFA01000001–BNFA01000004
Zam_UTH_05	SAMD00243951	SAMD00243951	BNFB01000001–BNFB01000002
Zam_UTH_06	SAMD00243952	SAMD00243952	BNFC01000001–BNFC01000010
Zam_UTH_07	SAMD00243953	SAMD00243953	BNFD01000001–BNFD01000004
Zam_UTH_08	SAMD00243954	SAMD00243954	BNFE01000001–BNFE01000009
Zam_UTH_09	SAMD00243955	SAMD00243955	BNFF01000001–BNFF01000007
Zam_UTH_10	SAMD00243956	SAMD00243956	BNFG01000001–BNFG01000003
Zam_UTH_11	SAMD00243957	SAMD00243957	BNFH01000001–BNFH01000004
Zam_UTH_12	SAMD00243958	SAMD00243958	BNFI01000001–BNFI01000013
Zam_UTH_13	SAMD00243959	SAMD00243959	BNFJ01000001–BNFJ01000003
Zam_UTH_15	SAMD00243960	SAMD00243960	BNFK01000001–BNFK01000003
Zam_UTH_17	SAMD00243961	SAMD00243961	BNFL01000001–BNFL01000010
Zam_UTH_18	SAMD00243962	SAMD00243962	BNFM01000001–BNFM01000001
Zam_UTH_20	SAMD00243963	SAMD00243963	BNFN01000001–BNFN01000007
Zam_UTH_21	SAMD00243964	SAMD00243964	BNFO01000001–BNFO01000003
Zam_UTH_22	SAMD00243965	SAMD00243965	BNFP01000001–BNFP01000010
Zam_UTH_23	SAMD00243966	SAMD00243966	BNFQ01000001–BNFQ01000005
Zam_UTH_24	SAMD00243967	SAMD00243967	BNFR01000001–BNFR01000011
Zam_UTH_25	SAMD00243968	SAMD00243968	BNFS01000001–BNFS01000011
Zam_UTH_26	SAMD00243969	SAMD00243969	BNFT01000001–BNFT01000003
Zam_UTH_27	SAMD00243970	SAMD00243970	BNFU01000001–BNFU01000004
Zam_UTH_28	SAMD00243971	SAMD00243971	BNFV01000001–BNFV01000003
Zam_UTH_29	SAMD00243972	SAMD00243972	BNFW01000001–BNFW01000015
Zam_UTH_30	SAMD00243973	SAMD00243973	BNFX01000001–BNFX01000005
Zam_UTH_31	SAMD00243974	SAMD00243974	BNFY01000001–BNFY01000010
Zam_UTH_32	SAMD00243975	SAMD00243975	BNFZ01000001–BNFZ01000012
Zam_UTH_33	SAMD00243976	SAMD00243976	BNGA01000001–BNGA01000004
Zam_UTH_34	SAMD00243977	SAMD00243977	BNGB01000001–BNGB01000016
Zam_UTH_36	SAMD00243978	SAMD00243978	BNGC01000001–BNGC01000015
Zam_UTH_37	SAMD00243979	SAMD00243979	BNGD01000001–BNGD01000006
Zam_UTH_38	SAMD00243980	SAMD00243980	BNGE01000001–BNGE01000010
Zam_UTH_39	SAMD00243981	SAMD00243981	BNGF01000001–BNGF01000004
Zam_UTH_40	SAMD00243982	SAMD00243982	BNGG01000001–BNGG01000003
Zam_UTH_41	SAMD00243983	SAMD00243983	BNGH01000001–BNGH01000016
Zam_UTH_42	SAMD00243984	SAMD00243984	BNGI01000001–BNGI01000015
Zam_UTH_43	SAMD00243985	SAMD00243985	BNGJ01000001–BNGJ01000007
Zam_UTH_44	SAMD00243986	SAMD00243986	BNGK01000001–BNGK01000008
Zam_UTH_45	SAMD00243987	SAMD00243987	BNGL01000001–BNGL01000008
Zam_UTH_46	SAMD00243988	SAMD00243988	BNGM01000001–BNGM01000014
Zam_UTH_47	SAMD00243989	SAMD00243989	BNGN01000001–BNGN01000013
Zam_UTH_48	SAMD00243990	SAMD00243990	BNGO01000001–BNGO01000017
Zam_UTH_50	SAMD00243991	SAMD00243991	BNGP01000001–BNGP01000009
Zam_UTH_51	SAMD00243992	SAMD00243992	BNGQ01000001–BNGQ01000015

## Conclusion

We characterized AMR phenotypes and genotypes of cefotaxime-resistant clinical *Enterobacteriaceae* and noted that the main mode of *bla*_CTX-M_ transmission was through the spread of clones of a few resilient STs. In one *E. cloacae* and three *E. coli* strains, chromosomally-integrated IS*Ecp1*-*bla*_CTX-M-15_ transposition unit existed on large insertions that concurrently harbored diverse AMR genes and originated from plasmids. Our findings suggest that IS*Ecp1* facilitates chromosomal integration of *bla*_CTX-M-15_-associated MDR determinants in diverse *Enterobacteriaceae* species. Stable maintenance of these determinants on chromosomes may promote the propagation of MDR clones among multiple *Enterobacteriaceae* species, jeopardizing the treatment efficacy of available antimicrobials.

## Supplementary Information

**Additional file 1.** Supplementary tables and figures (except Fig. S5).

**Additional file 2.** Figure S5.

## Data Availability

All raw reads have been deposited in the DDBJ under the DRA accession number, DRA010721. The nearly complete genome sequences have been deposited in the GenBank under the BioProject identifier PRJDB10450. Individual accession numbers for each strain are provided in Table 2.
