# Supplementary material for: Novel chromosomal insertions of ISEcp1-blaCTX-M-15 and diverse antimicrobial resistance genes in Zambian clinical isolates of Enterobacter cloacae and Escherichia coli
Source: Antimicrob Resist Infect Control. 2021 May 10;10:79. doi: 10.1186/s13756-021-00941-8 (PMC8111917; doi:10.1186/s13756-021-00941-8)
Supplement: Supplementary file 1 — Additional file 1. Supplementary tables and figures (except Fig. S5). [file 13756_2021_941_MOESM1_ESM.pdf]

Supplementary tables and figures

Supplementary Table 1. Antimicrobials used in this study

Antimicrobial	Class	Solvent	S	I	R
Ampicillin sodium	β-lactam	DW	≤ 8	16	≥ 32
Cefotaxime sodium	β-lactam	DW	≤ 1	2	≥ 4
Chloramphenicol	Amphenicol	100% ethanol	≤ 8	16	≥ 32
Ciprofloxacin	Quinolone	0.1 M NaOH	≤ 0.25	0.5	≥ 1
Colistin sulfate	Polymyxin	DW	≤ 2	-	≥ 4
Doxycycline hyclate	Tetracycline	DW	≤ 4	8	≥ 16
Gentamicin sulfate salt	Aminoglycoside	DW	≤ 4	8	≥ 16
Imipenem monohydrate	Carbapenem	1 M MOPS	≤ 1	2	≥ 4
Nalidixic acid free acid	Quinolone	0.1 M NaOH	≤ 16	-	≥ 32
Nitrofurantoin	Nitrofuran	DMF	≤ 32	64	≥ 128

NB; All antimicrobials were purchased from Sigma-Aldrich®. LB broth (Difco™) was used as diluent in all cases.

DMF = dimethyl formamide

DW = distilled water

MOPS = (3-(N-morpholino)propanesulfonic acid), pH 6.8

S = susceptible, I = intermediate, R = resistant

Supplementary Table 2. Primers used for amplification of chromosome/plasmid junctions

Name	Abbreviation	Sequence
Zam_UTH_41_jnc_1_inner_For	F1	GGCAGCCAATGAATCCGC
Zam_UTH_41_jnc_2_inner_Rev	R1	CGCAACAGGTCTTCTATCGACG
Zam_UTH_43_jnc_1_For	F2	CCAGGGATTTTCTACGGCAGG
Zam_UTH_43_jnc_1_Rev	R2	CGGACTCATTCCTCTCAGGATC
Zam_UTH_43_jnc_2_For	F3	GCTGCTGTGCAAAAAACAAGAC
Zam_UTH_43_jnc_2_Rev	R3	CGATGCTAAGCCATTTGCCTG
Str_18_jnc1_innermost_For	F4	CTGCCAGTCCCAGCACTTTG
Str_18_jnc1_inner_Rev	R4	GGCCTCAACACGAATGTCAT
Str_18_jnc2_inner_For	F5	TGCATTCTCAAGGAGCAGAA
Str_18_jnc2_innermost_Rev_1	R5	GTTCGTCAGGCTTTTTCTGGTG
Zam_UTH_42_jnc_1_For	F6	GCAAGAGGATAAACCGTCGGG
Zam_UTH_42_jnc_1_outer_Rev	R6	CCACACCCAGTCTGCCTCC
Zam_UTH_42_jnc_2_For	F7	GCACAACATGGGGGATCATG
Zam_UTH_42_jnc_2_Rev	R7	GGCATATTGCTTTGTGGTGGTG
Zam_UTH_44_jnc_1_For	F8	GTTACCCCGGCGTAGAGG
Zam_UTH_44_jnc_1_Rev	R8	GACCTGGACGTTGTGCTGAAG
Zam_UTH_44_jnc_2_For	F9	GGGCACTATTCATGCGTCAG
Zam_UTH_44_jnc_2_Rev	R9	GACGTTGTGCGCCAGTTC

Supplementary Table 3. Primers used for verification of *bla*$_{CTX-M}$ allele in seven strains

Strain	Primer	Sequence	Size (bp)
Zam_UTH_03	Zam_UTH_03_For	CGTATCAGCGCTGCATGC	1730
	Zam_UTH_03_Rev	CTTTGCAACAGTGCCCCG	
Zam_UTH_06	Zam_UTH_06_For	GAGTGTTGCTCTGTGGATAAC	1324
	Zam_UTH_06_Rev	GTCTGCCTCGTGAAGAAGGTG	
Zam_UTH_22	Zam_UTH_22_For	GGAGCCACGGTTGATGAGG	1230
	Zam_UTH_22_Rev	GCTCTGTGGATAACTTGCAGAG	
Zam_UTH_25	Zam_UTH_25_For	CAGCGTAGCGGAACGTTC	1248
	Zam_UTH_25_Rev	GGATTGACCGTATTGGGAGTTTG	
Zam_UTH_26	Zam_UTH_26_For	CGGAAAACTATCCGTACAAGGG	1456
	Zam_UTH_26_Rev	CCGGCGGAAACAATGAGAAAAC	
Zam_UTH_28	Zam_UTH_28_For	CACCGACATTACACCGGGC	1458
	Zam_UTH_28_Rev	GCTCTGTGGATAACTTGCAGAG	
Zam_UTH_34	Zam_UTH_34_For	CGCCTCGCCACACTAATC	1397
	Zam_UTH_34_Rev	CTGATGTAACACGGATTGACC	

Supplementary Table 4. AMR genes detected

Target drug class	AMR genes detected
β-lactam	bla_{TEM-1}, bla_{TEM-84}, bla_{SHV-11}, $bla_{SHV-121}$, bla_{SHV-28}, bla_{OXA-1}, $bla_{CTX-M-14}$, $bla_{CTX-M-15}$, $bla_{CTX-M-27}$, $bla_{CTX-M-55}$, bla_{ACT}, bla_{CMY-2}
Aminoglycoside	*aph(3')-Ia, aph(3')-IIa, aph(3")-Ib, aph(6)-Id, aac(3)-IIa, aac(3)-IId, aac(6')-Ib4, aac(6')-Ib-cr5*[b]*, aadA1, aadA2, aadA5,*
Streptothricin	*sat2*
Trimethoprim	*dfrA1, dfrA5, dfrA12, dfrA14, dfrA17*
Tetracycline	*tet(A), tet(B), tet(C), tet(D), tet(M)*
Chloramphenicol	*cmlA1, catA1, catA2, catB3, floR*
Sulfonamide	*sul1, sul2, sul3*
Fosfomycin	*fosA, fosA3*
Macrolide	*erm(B), mph(A)*
Quinolone	*oqxA, oqxB, oqxB19, qnrB1, qnrB19, qnrS1, qnrS13,*
Disinfectant	*qacE, qacL, qacEdelta1*
Bleomycin	*bleO, ble*

[b]aminoglycoside and quinolone

Supplementary Table 5. Prediction of phenotype from AMR genes.

**GEN**	*aph(3)-Ia*	*aph(3)-IIa*	*aph(3'')-Ib*	*aph(6)-Id*	*aac(3)-IIa*	*aac(3)-IId*
R	10	1	28	28	14	13
S	0	0	3	3	0	0
Total	10	1	31	31	14	13
**PPV (%)**	**100**	**100**	**90.3**	**90.3**	**100**	**100**

**GEN**	*aac(6')-Ib4*	*aac(6')-Ib-cr5*	*aadA1*	*aadA2*	*aadA5*
R	1	16	12	10	19
S	0	2	0	0	1
Total	1	18	12	10	20
**PPV (%)**	**100**	**88.9**	**100**	**100**	**95**

**CIP**	*oqxA*	*oqxB*	*oqxB19*	*qnrB1*	*qnrB19*	*qnrS1*	*qnrS13*
R	11	5	5	7	2	9	2
S	0	0	0	0	0	1	0
Total	11	5	5	7	2	10	2
**PPV (%)**	**100**	**100**	**100**	**100**	**100**	**90**	**100**

**NAL**	*oqxA*	*oqxB*	*oqxB19*	*qnrB1*	*qnrB19*	*qnrS1*	*qnrS13*
R	11	5	5	7	2	9	2
S	0	0	0	0	0	1	0
Total	11	5	5	7	2	10	2
**PPV (%)**	**100**	**100**	**100**	**100**	**100**	**90**	**100**

**DOX**	*tet(A)*	*tet(B)*	*tet(C)*	*tet(D)*	*tet(M)*
R	24	9	1	3	9
S	0	0	0	0	0
Total	24	9	1	3	9
**PPV (%)**	**100**	**100**	**100**	**100**	**100**

**CHL**	*cmlA1*	*catA1*	*catA2*	*catB3*
R	8	3	2	6
S	2	0	0	9
Total	10	3	2	15
**PPV (%)**	**80**	**100**	**100**	**42.9**

GEN = gentamicin, CIP = ciprofloxacin, NAL = nalidixic acid, DOX = doxycycline, CHL = chloramphenicol

R = resistant, S = susceptible

Supplementary Table 6: Prediction of phenotype from AMR genes in strains with chromosomal *bla*$_{CTX-M}$

**Strain ID**	**Gene**	**Location**[c]	**Drug class affected**	**Phenotype**[d]
Zam_UTH_26	*dfrA1*	Chromosome	Trimethoprim	NT
	sat2	Chromosome	Streptothricin	NT
	aadA1	Chromosome	Aminoglycoside	R
	bla$_{EC}$	Chromosome	β-lactam	R
	sul2	Chromosome	Sulfonamide	NT
	bla$_{TEM-1}$	Chromosome	β-lactam	R
	tet(D)	Chromosome	Tetracycline	R
	bla$_{CTX-M-14}$	ChromIns	β-lactam	R
Zam_UTH_41	*sul2*	Chromosome	Sulfonamide	NT
	aph(3'')-Ib	Chromosome	Aminoglycoside	R
	aph(6)-Id	Chromosome	Aminoglycoside	R
	bla$_{TEM-1}$	Chromosome	β-lactam	R
	bla$_{CTX-M-14}$	ChromIns	β-lactam	R
	dfrA1	Chromosome	Trimethoprim	NT
	sat2	Chromosome	Streptothricin	NT
	bla$_{EC}$	Chromosome	β-lactam	R
	aadA1	Chromosome	Aminoglycoside	R
	bla$_{CTX-M-15}$	Plasmid	β-lactam	R
Zam_UTH_43	*aac(3)-II*	Plasmid	Aminoglycoside	R

	aac(3)-II	Plasmid	Aminoglycoside	R
	tet(A)	Plasmid	Tetracycline	R
	aph(6)-Id	Plasmid	Aminoglycoside	R
	aph(3'')-Ib	Plasmid	Aminoglycoside	R
	sul2	Plasmid	Sulfonamide	NT
	bla$_{TEM-1}$	Plasmid	β-lactam	R
	mph(A)	Plasmid	Macrolide	NT
	sul1	Plasmid	Sulfonamide	NT
	qacEdelta1	Plasmid	Disinfectant	NT
	aadA5	Plasmid	Aminoglycoside	
	dfrA17	Plasmid	Trimethoprim	NT
	bla$_{EC}$	Chromosome	β-lactam	R
	bla$_{CTX-M-15}$	ChromIns	β-lactam	R
	bla$_{OXA-1}$	Chromosome	β-lactam	R
	aac(6')-Ib-cr5	Chromosome	Aminoglycoside/Quinolone	R/R
Zam_UTH_18	*bla*$_{EC}$	Chromosome	β-lactam	R
	qnrS1	ChromIns	Quinolone	S
	bla$_{CTX-M-15}$	ChromIns	β-lactam	R
Zam_UTH_42	*bla*$_{EC}$	Chromosome	β-lactam	R
and	*aph(6)-Id*	Plasmid	Aminoglycoside	R
Zam_UTH_47	*dfrA14*	Plasmid	Trimethoprim	NT
	aph(3'')-Ib	Plasmid	Aminoglycoside	R

	sul2	Plasmid	Sulfonamide	NT
	tet(B)	Plasmid	Tetracycline	R
	dfrA17	Plasmid	Trimethoprim	NT
	aadA5	Plasmid	Aminoglycoside	R
	qacEdelta1	Plasmid	Disinfectant	NT
	sul1	Plasmid	Sulfonamide	NT
	mph(A)	Plasmid	Macrolide	NT
	catA1	Plasmid	Chloramphenicol	R
	bla$_{\text{CTX-M-15}}$	ChromIns	β-lactam	R
	aac(6')-Ib-cr5	ChromIns	Aminoglycoside/Quinolone	R/R
	bla$_{\text{OXA-1}}$	ChromIns	β-lactam	R
	catB3	ChromIns	Chloramphenicol	R
	aac(3)-IIa	ChromIns	Aminoglycoside	R
	bla$_{\text{TEM-1}}$	ChromIns	β-lactam	R
Zam_UTH_44	*oqxA*	Chromosome	Quinolone	R
	oqxB	Chromosome	Quinolone	R
	bla$_{\text{CTX-M-15}}$	ChromIns	β-lactam	R
	aac(3)-IIa	ChromIns	Aminoglycoside	R
	catB3	ChromIns	Chloramphenicol	R
	bla$_{\text{OXA-1}}$	ChromIns	β-lactam	R
	aac(6')-Ib-cr5	ChromIns	Aminoglycoside/Quinolone	R/R
	qnrB1	ChromIns	Quinolone	R
	dfrA14	ChromIns	Trimethoprim	NT

tet(A)	ChromIns	Tetracycline	R
fosA	Chromosome	Fosfomycin	NT
bla_{ACT}	Chromosome	β-lactam	R

[c]ChromIns = $bla_{\text{CTX-M}}$-carrying chromosomal insertion; [d]NT = Not tested

A

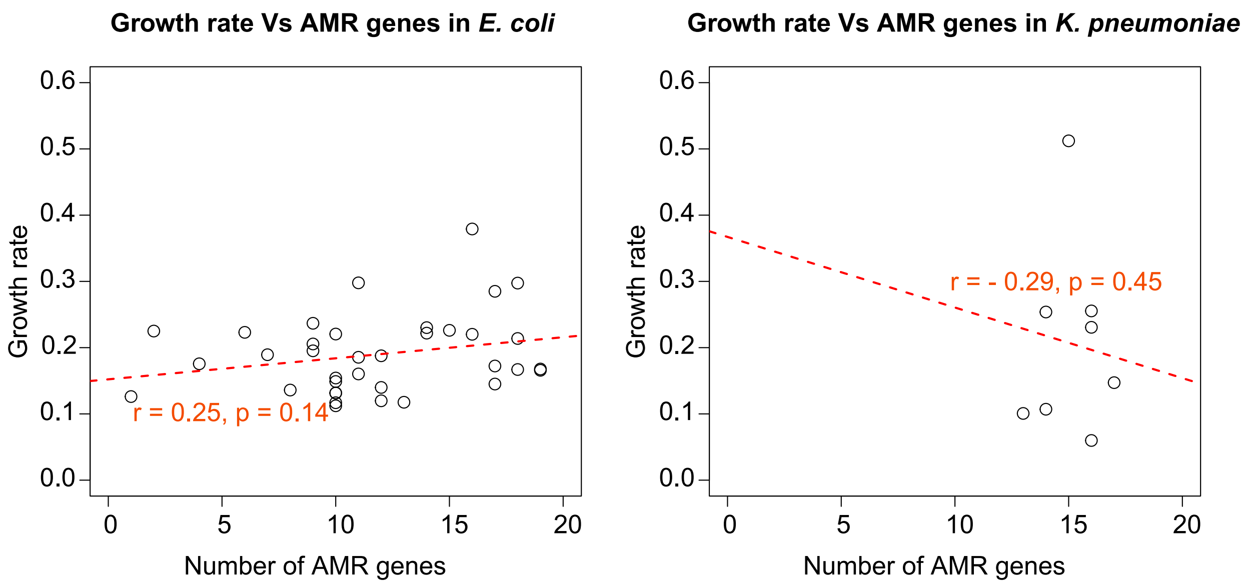

B

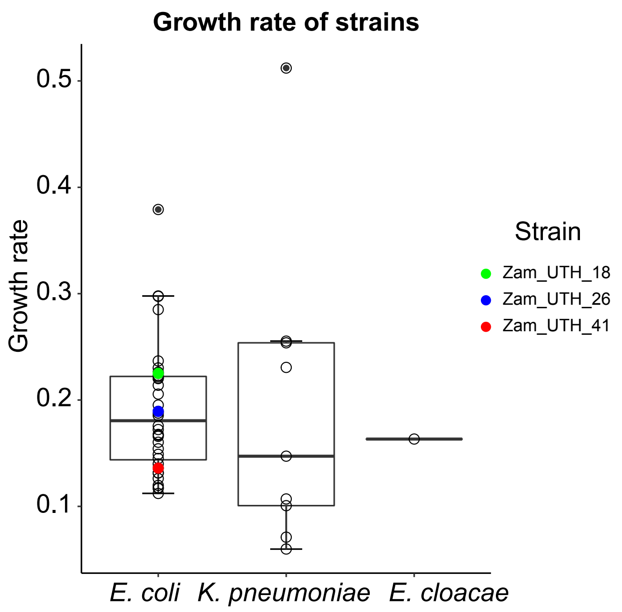

Supplementary Figure 1. Assessment of growth rate among strains

A. The analysis showed no significant correlation between growth rate and number of AMR genes. Left; *E. coli*. Right; *K. pneumoniae*. Red lines represent the linear regression models predicting growth rate using the number of AMR genes, while r is Pearson's correlation coefficient.

B. Zam_UTH_18 did not harbor any plasmid and grew at above the 75th percentile of the growth rate for *E. coli* strains in this study. Zam_UTH_26 had a growth rate above the

median growth rate for *E. coli* but a closely related strain, Zam_UTH_41, carried an additional *bla*$_{CTX\text{-}M\text{-}15}$ on an extra plasmid and grew at a rate lower than the 25th percentile.

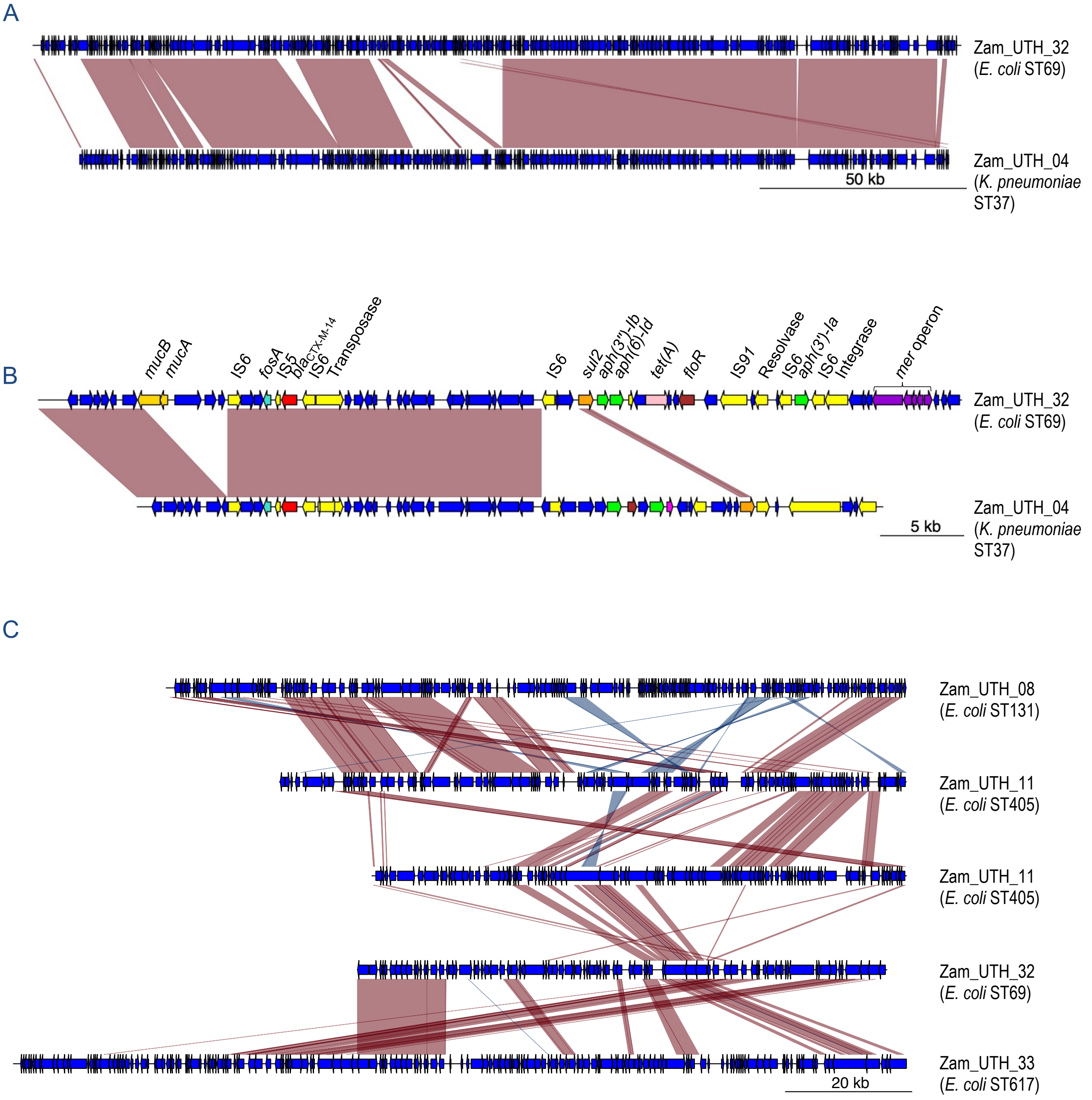

Supplementary Figure 2. Comparison of plasmid sequences among strains

A. There was over 80% nucleotide sequence homology between IncH plasmid in *E. coli* ST69 and IncH plasmid in *K. pneumoniae* ST37.

B. IncH plasmid in *E. coli* ST69 and IncH plasmid in *K. pneumoniae* ST37 both contained the *fosA*/IS*5*/*bla*$_{\text{CTX-M-14}}$/IS*6* gene cassette. IncH plasmid in *E. coli* ST69 also carried the *mucAB* operon as well as the *mer* operon.

C. There was low nucleotide sequence homology between IncF plasmids from *E. coli* strains belonging to different STs. Strains belonging to *E. coli* ST405 carried two IncF plasmids.

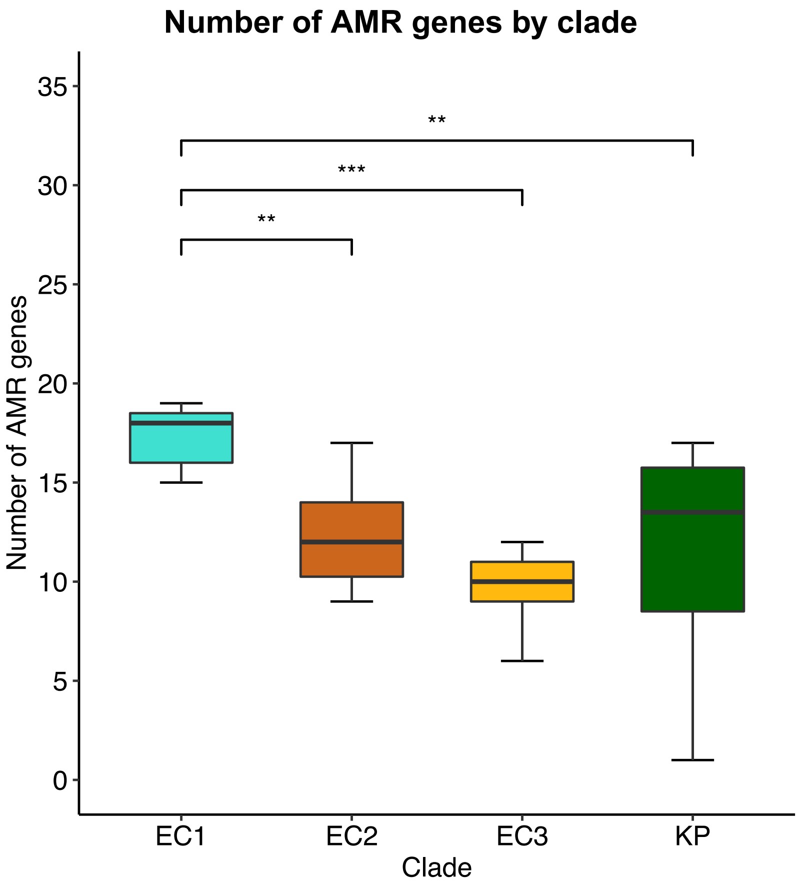

Supplementary Figure 3. Comparison of number of AMR genes among clades

The number of AMR genes in clade EC1 was significantly higher than what was observed in other clades.

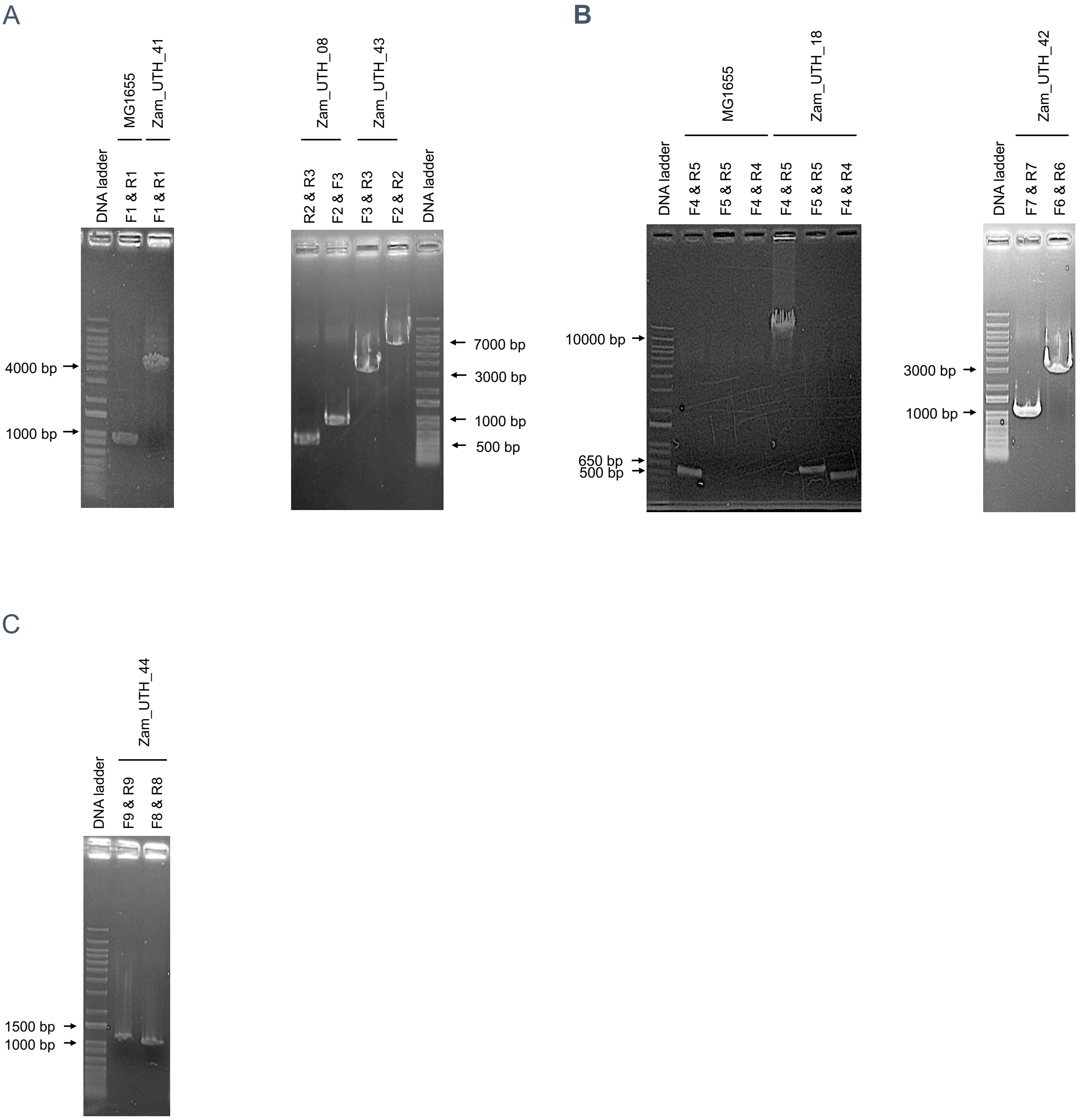

Supplementary Figure 4. Confirmation of chromosomal insertions

A. Short chromosomal insertions in *E. coli*. Left. PCR using the same primer set produced bands ~ 4 kb and ~ 1 kb in Zam_UTH_41 and *E. coli* MG1655, respectively. Right. PCR using primer sets F2/R2 and F3/R3 yielded products of sizes ~ 7 kb and ~ 3 kb, respectively in Zam_UTH_43, but primer sets F2/F3 and R2/R3 gave products around 850 bp and 500 bp, respectively in Zam_UTH_08 (control).

B. Large chromosomal insertions in *E. coli*. Left. PCR targeting the junctions between chromosome and plasmid produced amplicons between 500 bp and 650 bp in Zam_UTH_18, but no products were obtained in the reference strain *E. coli* MG1655. Moreover, when primers external to the insertion were used, a band larger than 10 kb was obtained in Zam_UTH_18 while the control strain *E. coli* MG1655 produced an amplicon between 500 bp and 650 bp. Right. PCR targeting junctions between chromosome and plasmid in Zam_UTH_42 produced bands of expected size. However, there was no control strain for comparison.

C. Large chromosomal insertion in *E. cloacae* (Zam_UTH_44). PCR targeting junctions between chromosome and plasmid gave bands of expected size, but there was no control strain for comparison.
