# Supplementary material for: Novel chromosomal insertions of ISEcp1-blaCTX-M-15 and diverse antimicrobial resistance genes in Zambian clinical isolates of Enterobacter cloacae and Escherichia coli
Source: Antimicrob Resist Infect Control. 2021 May 10;10:79. doi: 10.1186/s13756-021-00941-8 (PMC8111917; doi:10.1186/s13756-021-00941-8)
Supplement: Supplementary file 2 — Additional file 2. Figure S5. [file 13756_2021_941_MOESM2_ESM.pdf]

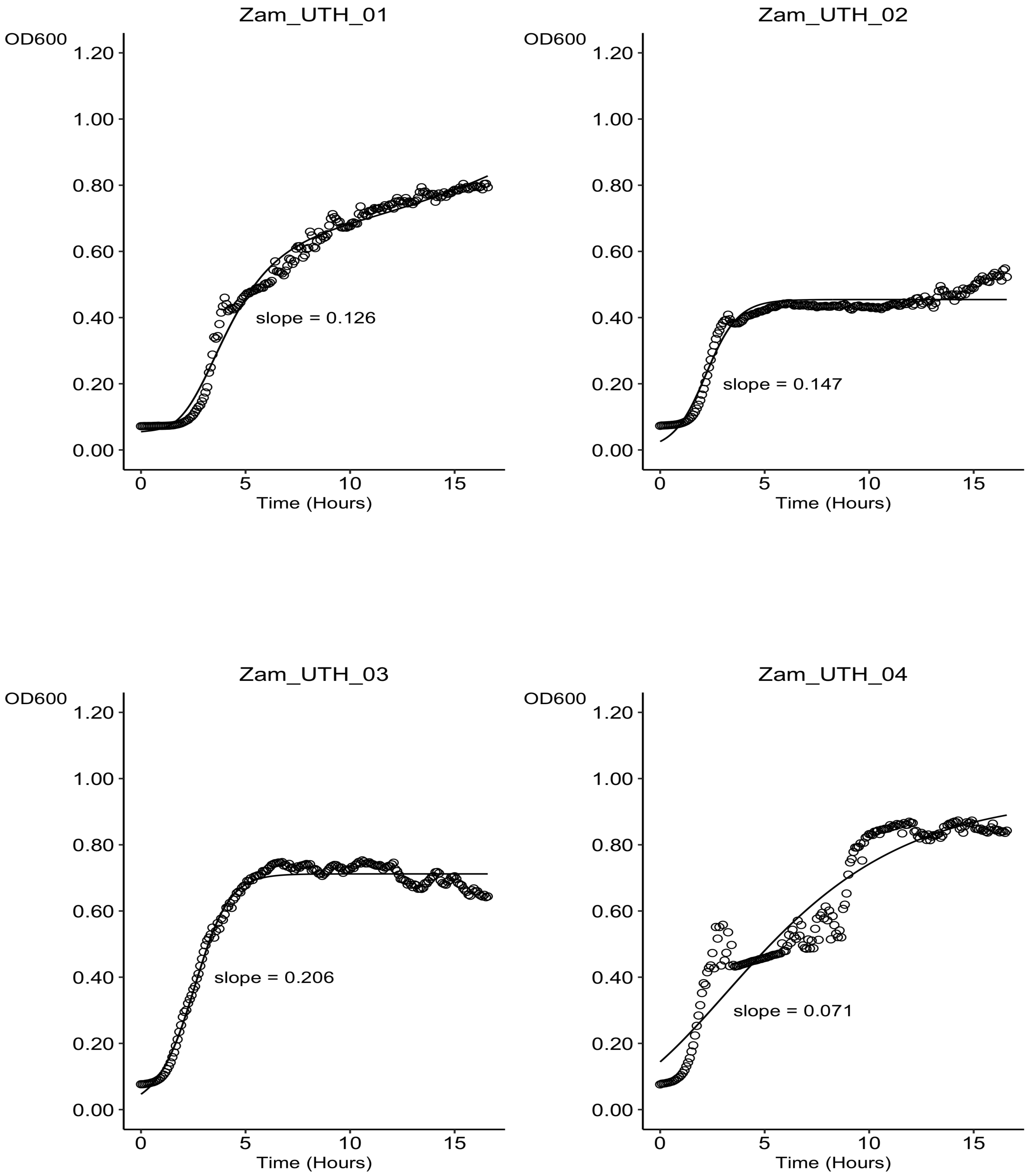
Zam_UTH_01
OD600
1.20
1.00
0.80
0.60
0.40
0.20
0.00
0
5
10
15
Time (Hours)
slope = 0.126
Zam_UTH_02
OD600
1.20
1.00
0.80
0.60
0.40
0.20
0.00
0
5
10
15
Time (Hours)
slope = 0.147
Zam_UTH_03
OD600
1.20
1.00
0.80
0.60
0.40
0.20
0.00
0
5
10
15
Time (Hours)
slope = 0.206
Zam_UTH_04
OD600
1.20
1.00
0.80
0.60
0.40
0.20
0.00
0
5
10
15
Time (Hours)
slope = 0.071

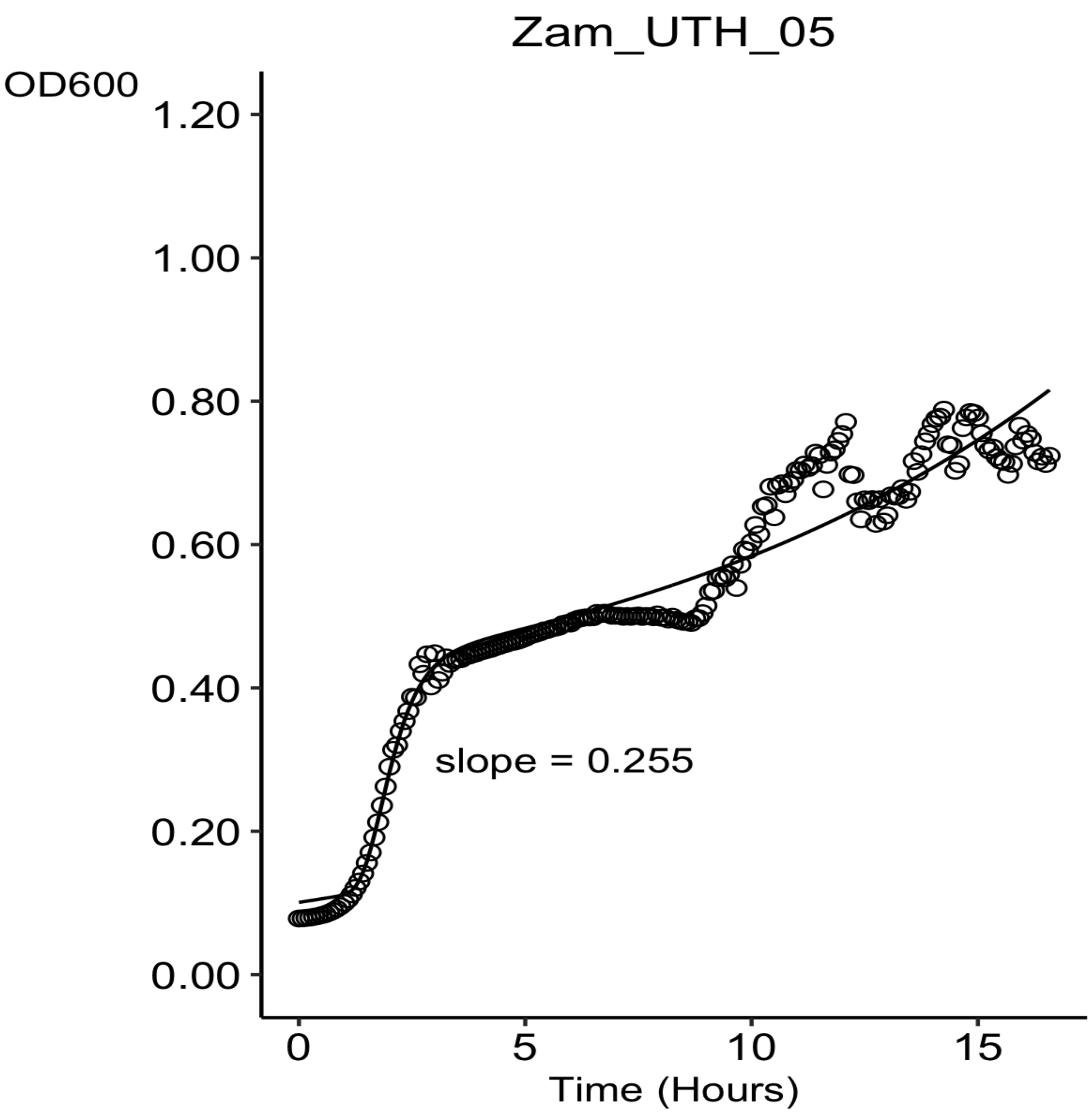
Zam_UTH_05
OD600
1.20
1.00
0.80
0.60
0.40
0.20
0.00
slope = 0.255
0
5
10
15
Time (Hours)

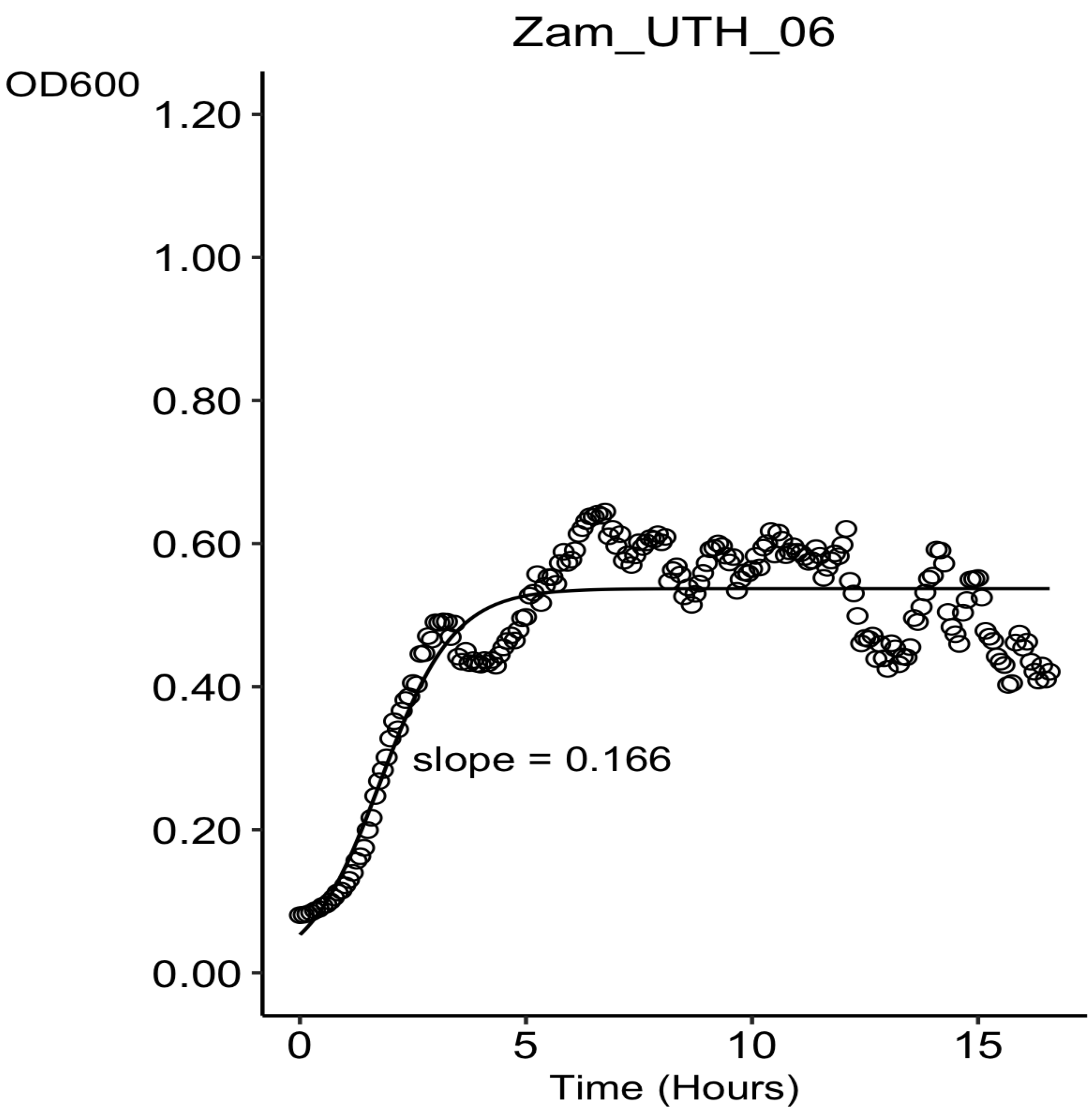
Zam_UTH_06
OD600
1.20
1.00
0.80
0.60
0.40
0.20
0.00
slope = 0.166
0
5
10
15
Time (Hours)

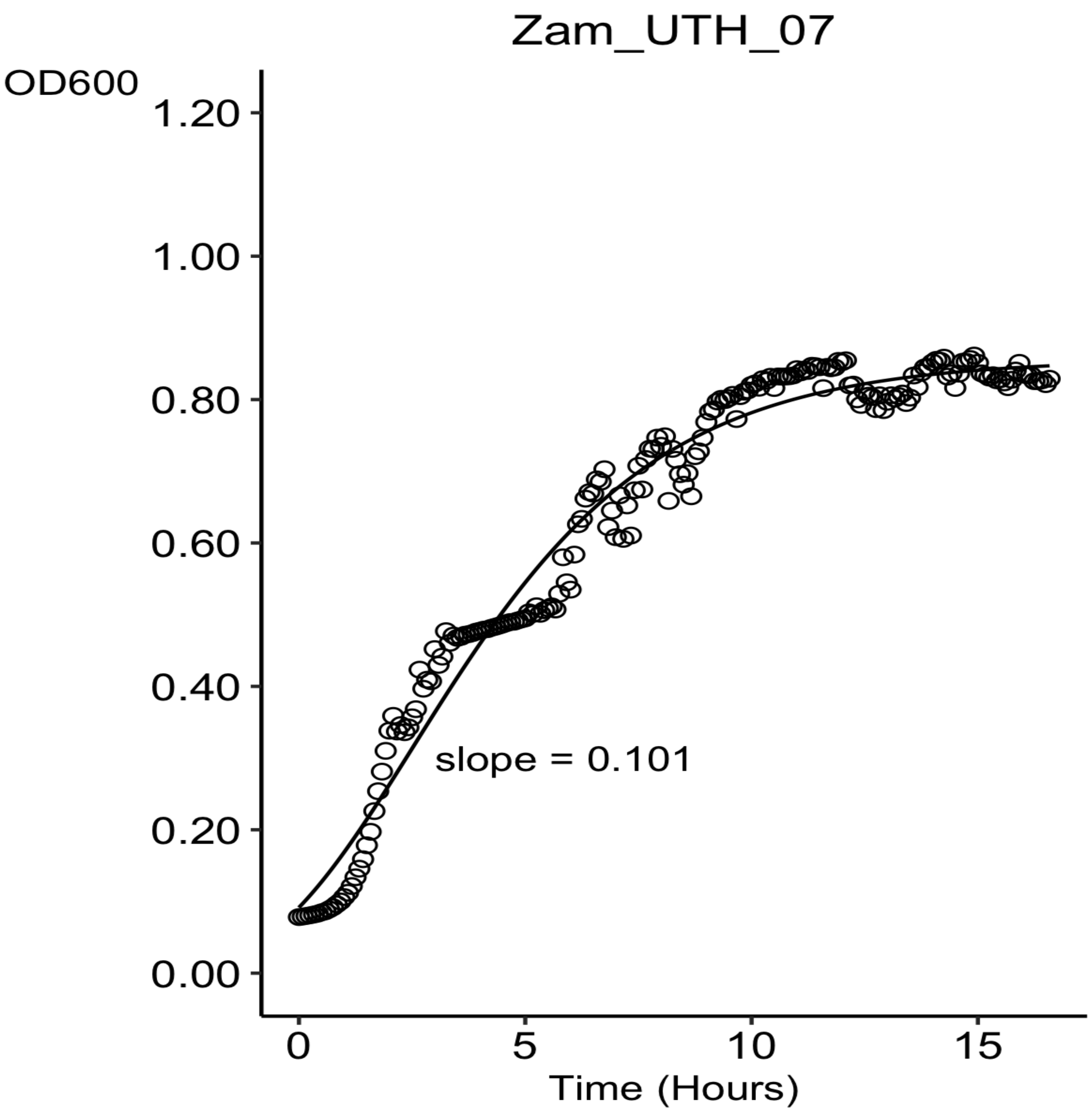
Zam_UTH_07
OD600
1.20
1.00
0.80
0.60
0.40
0.20
0.00
slope = 0.101
0
5
10
15
Time (Hours)

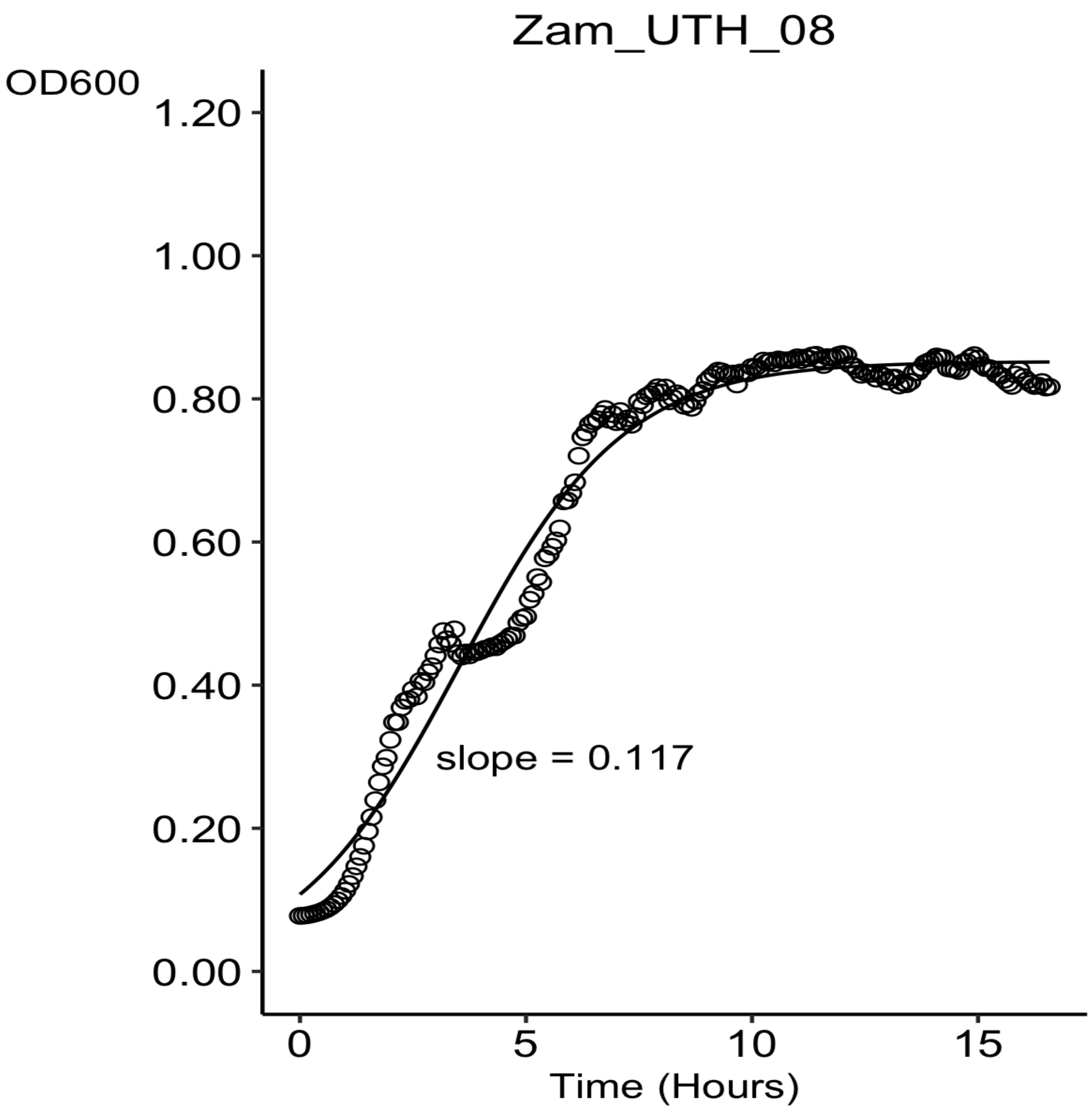
Zam_UTH_08
OD600
1.20
1.00
0.80
0.60
0.40
0.20
0.00
slope = 0.117
0
5
10
15
Time (Hours)

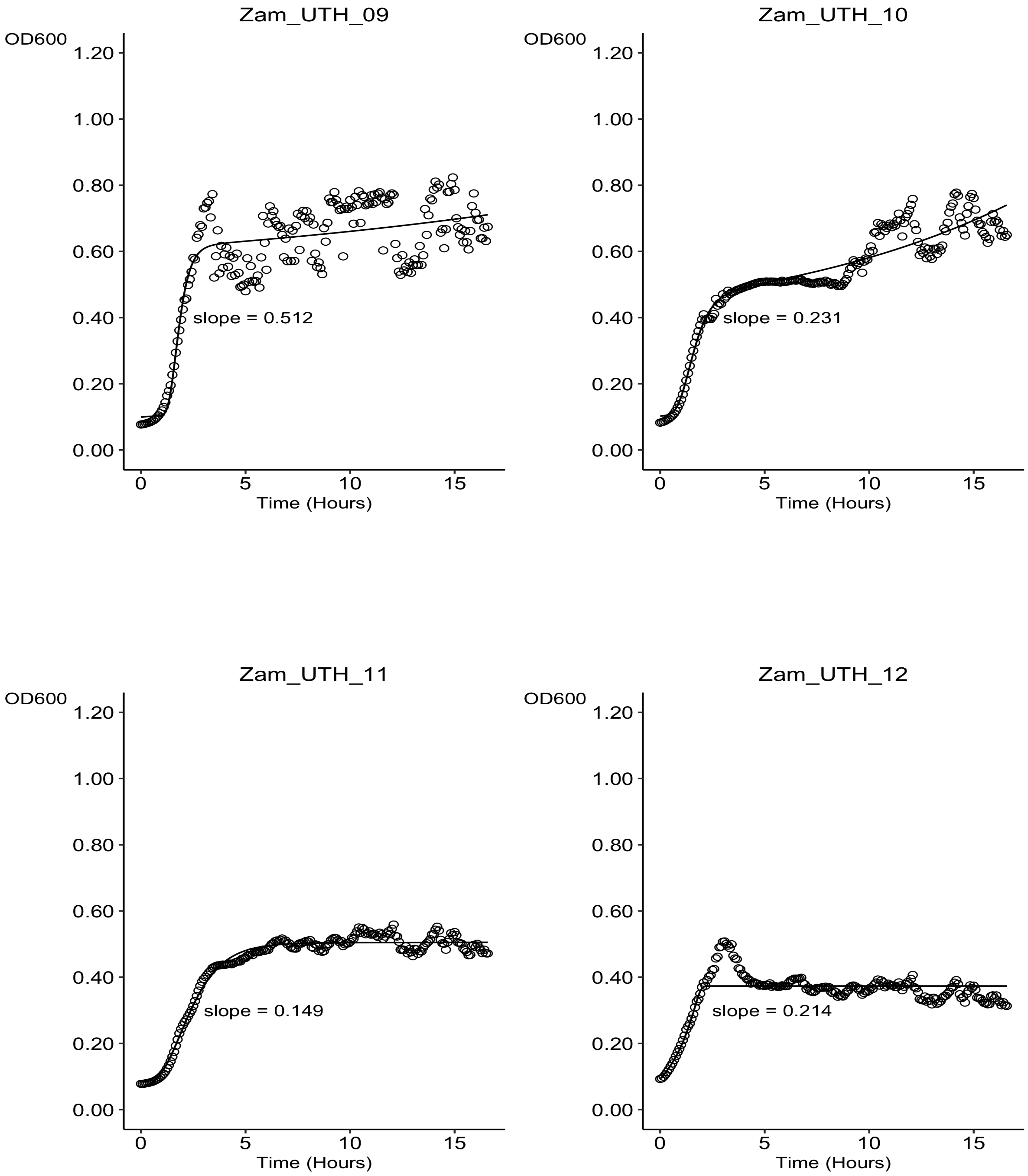

Zam_UTH_09
OD600
1.20
1.00
0.80
0.60
0.40
0.20
0.00
slope = 0.512
0
5
10
15
Time (Hours)
Zam_UTH_10
OD600
slope = 0.231
Time (Hours)
Zam_UTH_11
OD600
slope = 0.149
Time (Hours)
Zam_UTH_12
OD600
slope = 0.214
Time (Hours)

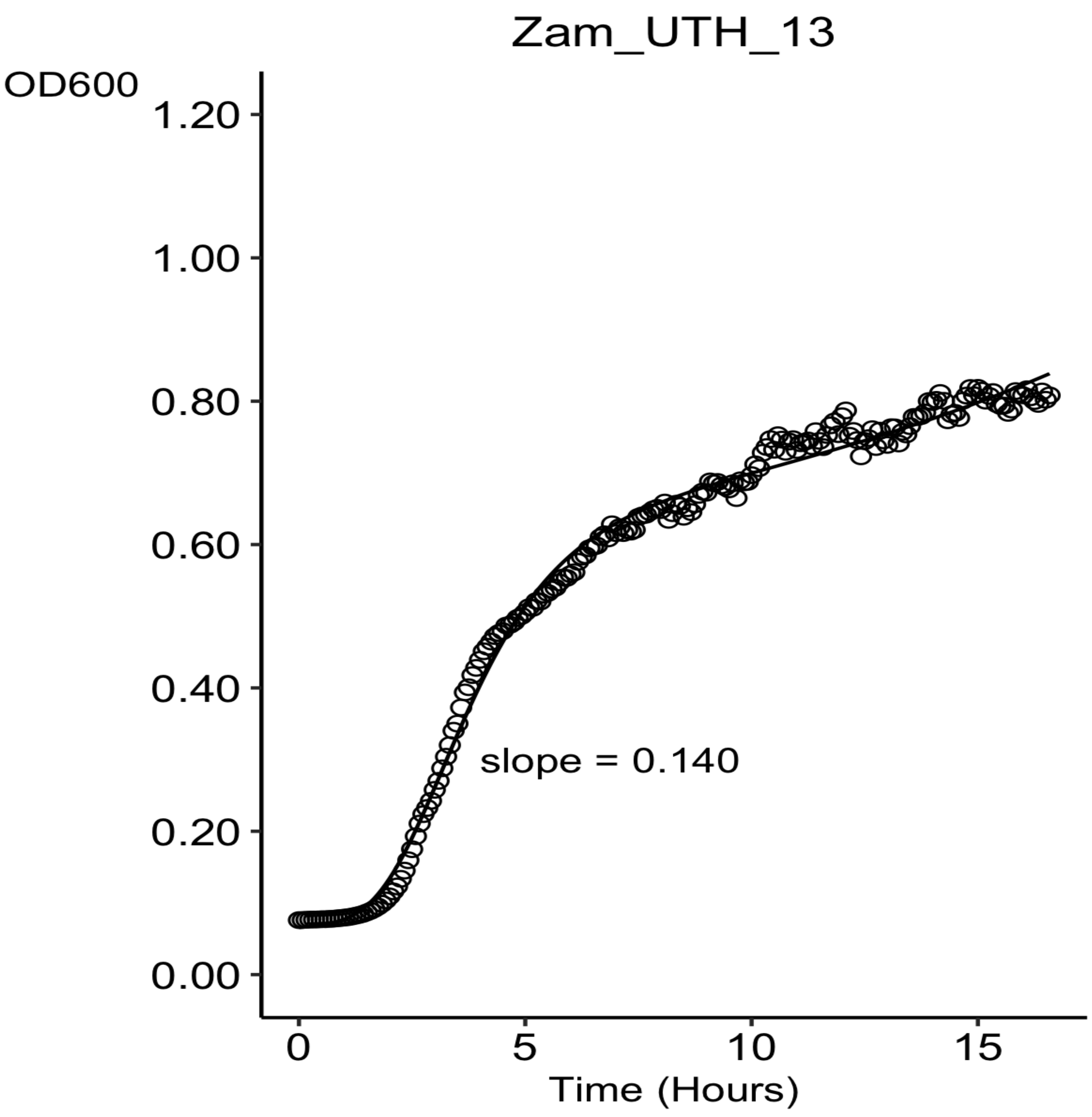
Zam_UTH_13
OD600
1.20
1.00
0.80
0.60
0.40
0.20
0.00
slope = 0.140
0
5
10
15
Time (Hours)

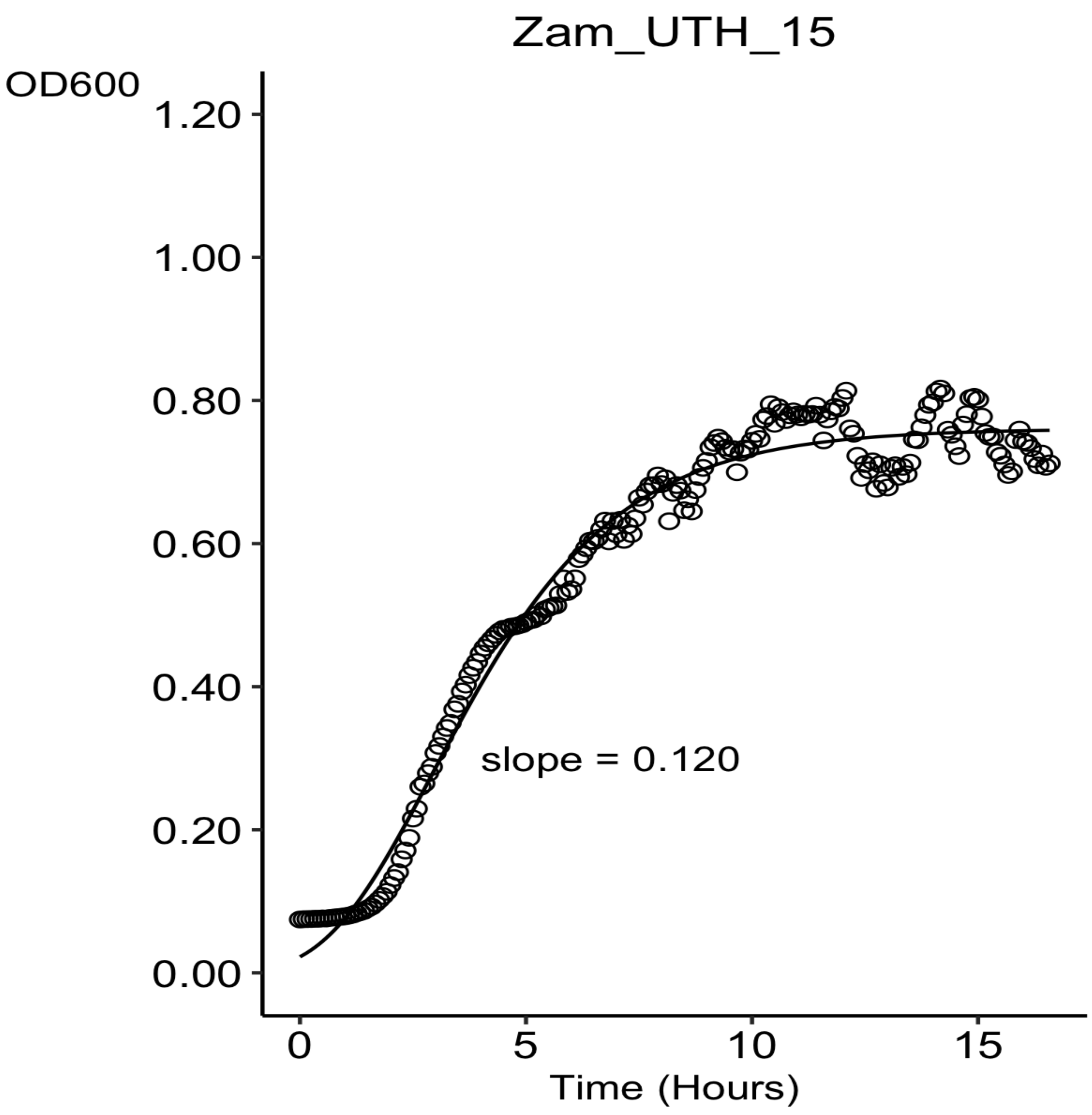
Zam_UTH_15
OD600
1.20
1.00
0.80
0.60
0.40
0.20
0.00
slope = 0.120
0
5
10
15
Time (Hours)

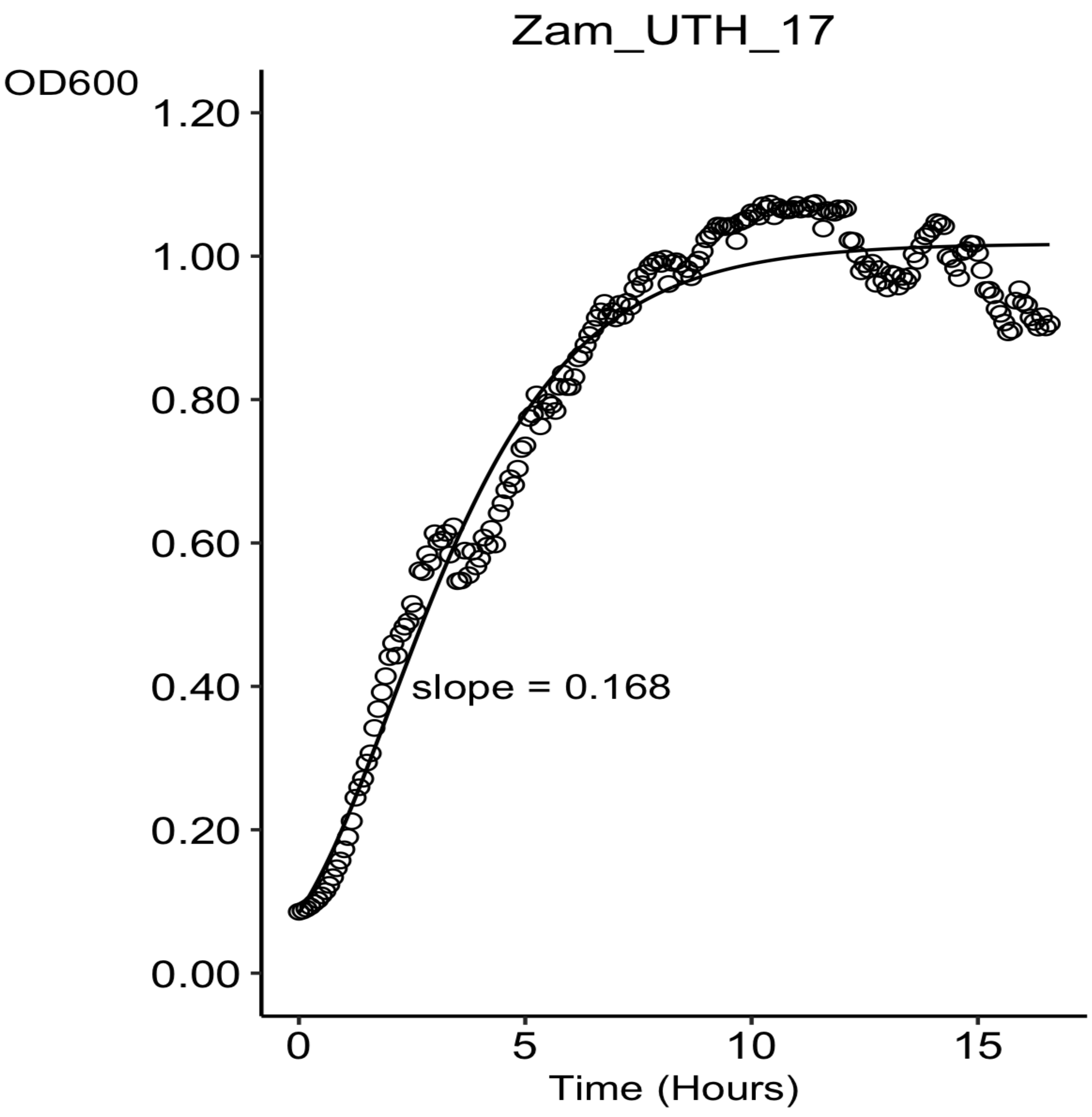
Zam_UTH_17
OD600
1.20
1.00
0.80
0.60
0.40
0.20
0.00
slope = 0.168
0
5
10
15
Time (Hours)

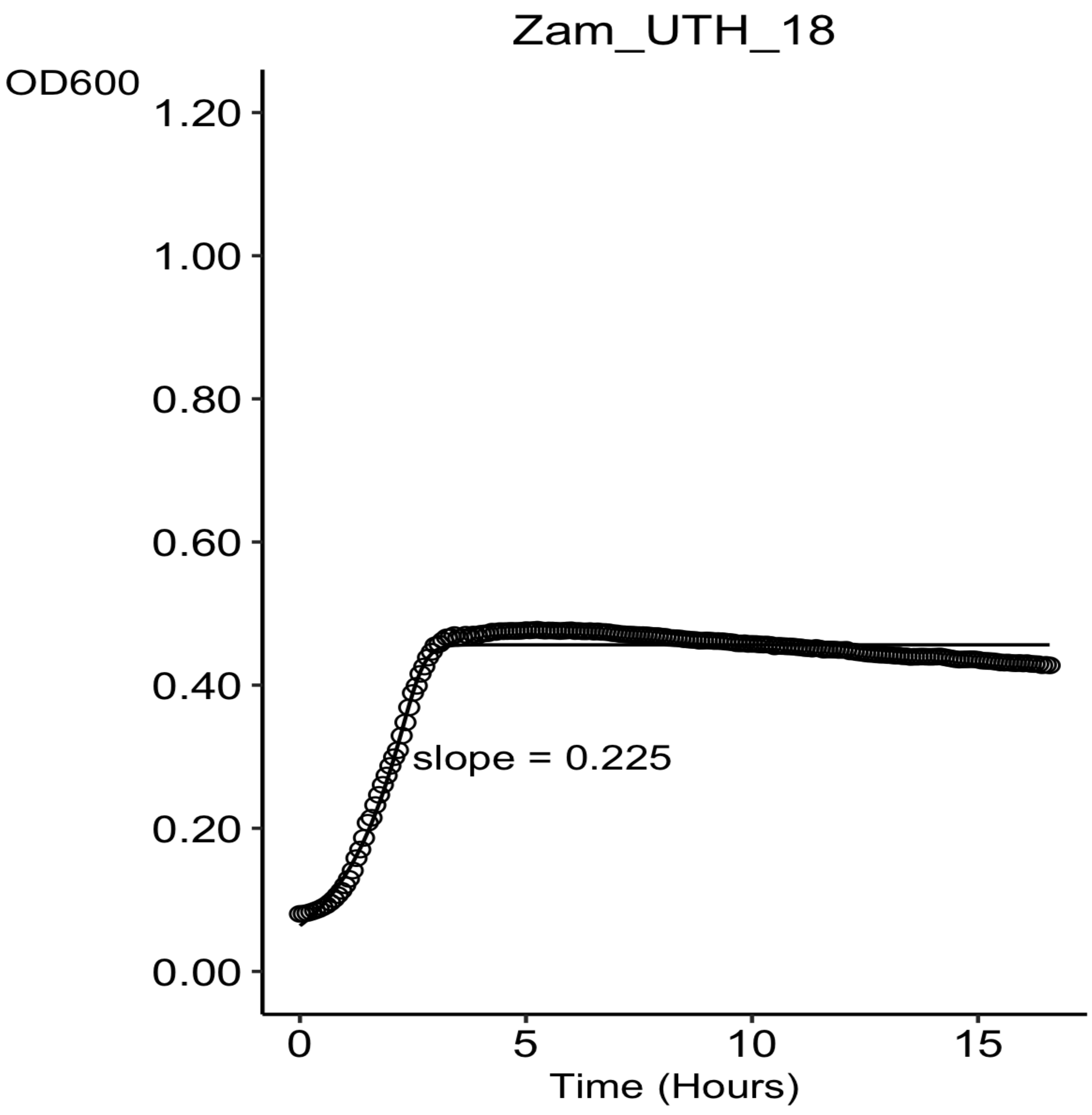
Zam_UTH_18
OD600
1.20
1.00
0.80
0.60
0.40
0.20
0.00
slope = 0.225
0
5
10
15
Time (Hours)

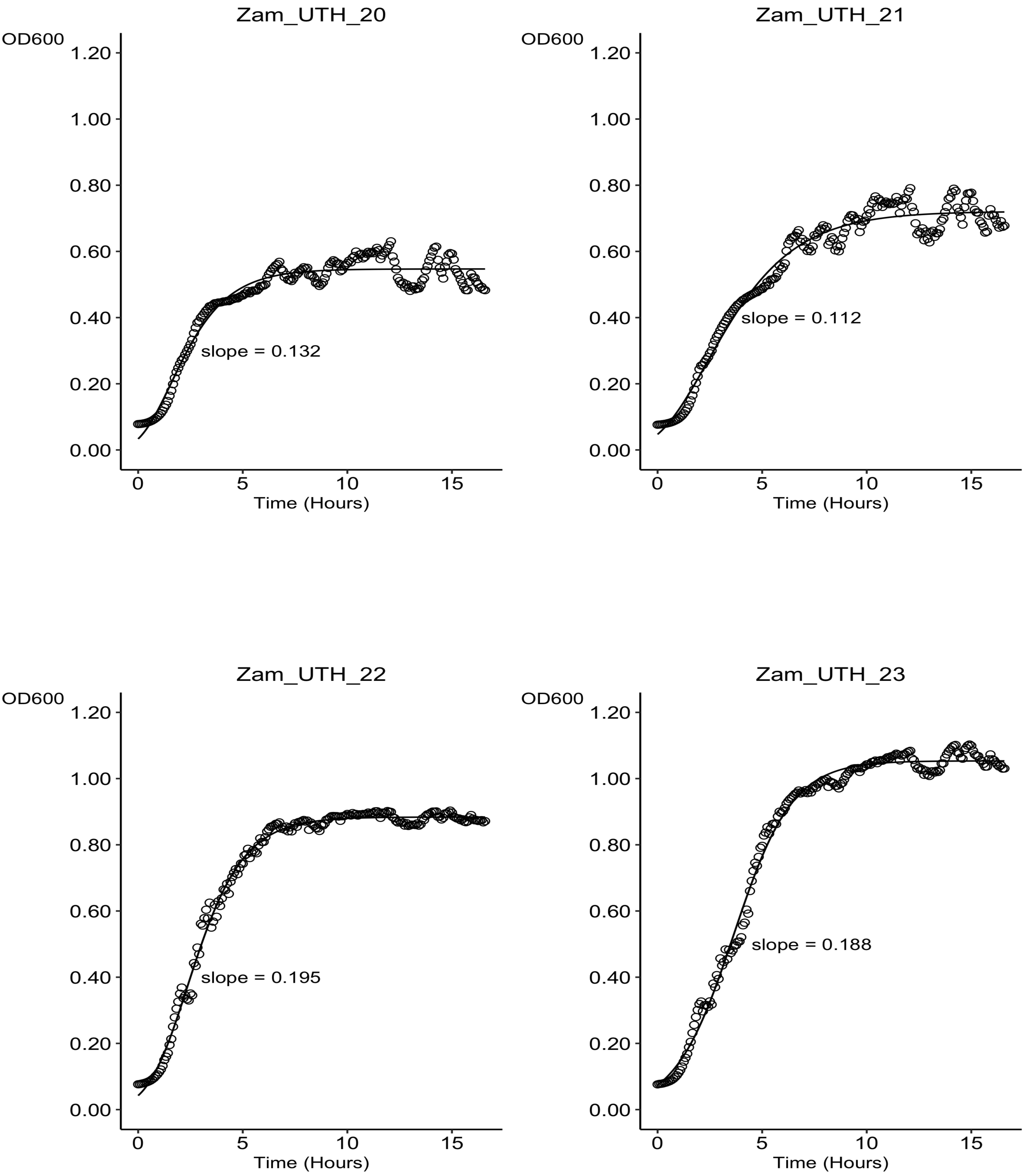

Zam_UTH_20
OD600
1.20
1.00
0.80
0.60
0.40
0.20
0.00
slope = 0.132
0
5
10
15
Time (Hours)
Zam_UTH_21
OD600
1.20
1.00
0.80
0.60
0.40
0.20
0.00
slope = 0.112
0
5
10
15
Time (Hours)
Zam_UTH_22
OD600
1.20
1.00
0.80
0.60
0.40
0.20
0.00
slope = 0.195
0
5
10
15
Time (Hours)
Zam_UTH_23
OD600
1.20
1.00
0.80
0.60
0.40
0.20
0.00
slope = 0.188
0
5
10
15
Time (Hours)

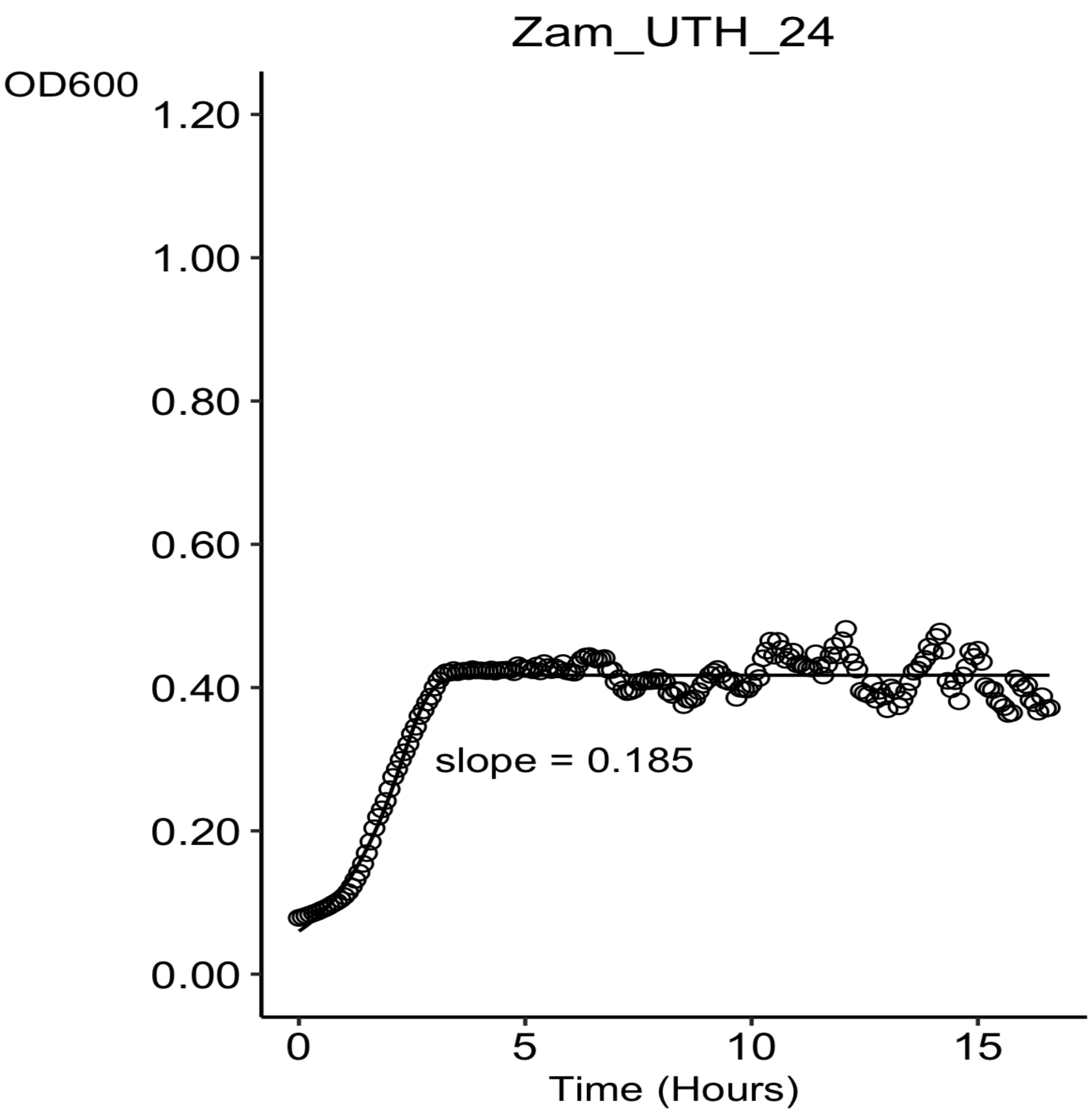
Zam_UTH_24
OD600
1.20
1.00
0.80
0.60
0.40
0.20
0.00
slope = 0.185
0
5
10
15
Time (Hours)

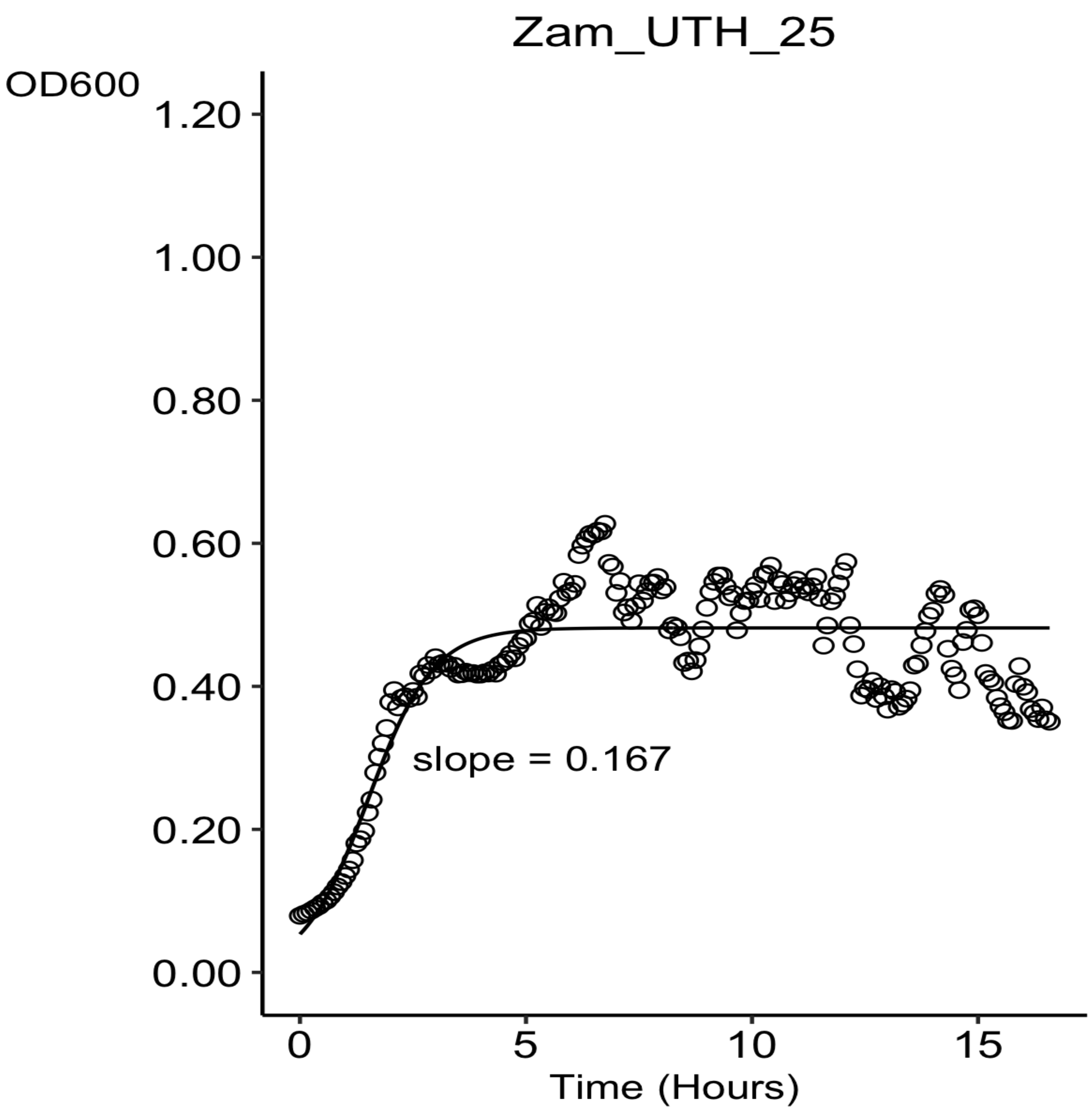
Zam_UTH_25
OD600
1.20
1.00
0.80
0.60
0.40
0.20
0.00
slope = 0.167
0
5
10
15
Time (Hours)

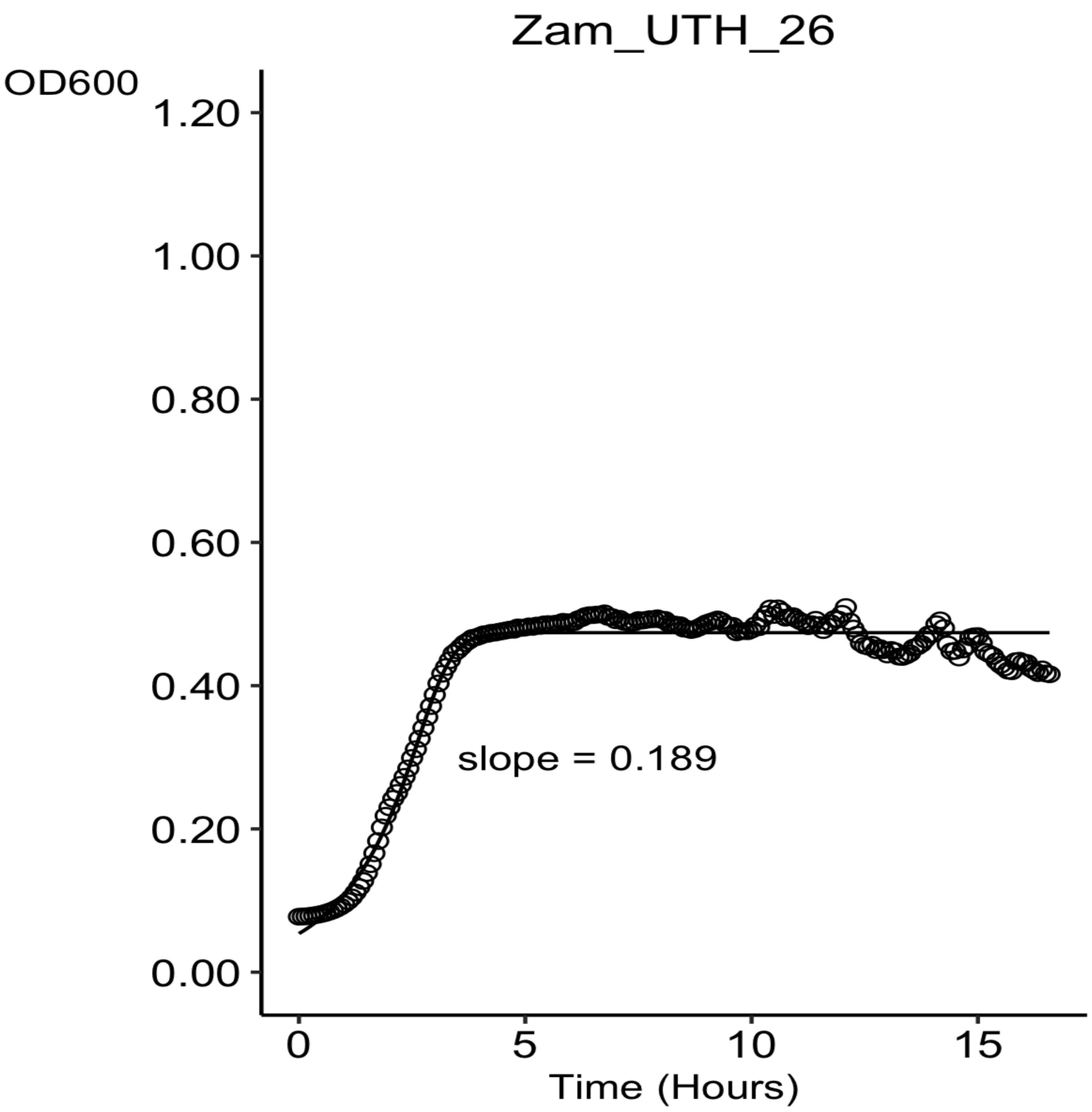
Zam_UTH_26
OD600
1.20
1.00
0.80
0.60
0.40
0.20
0.00
slope = 0.189
0
5
10
15
Time (Hours)

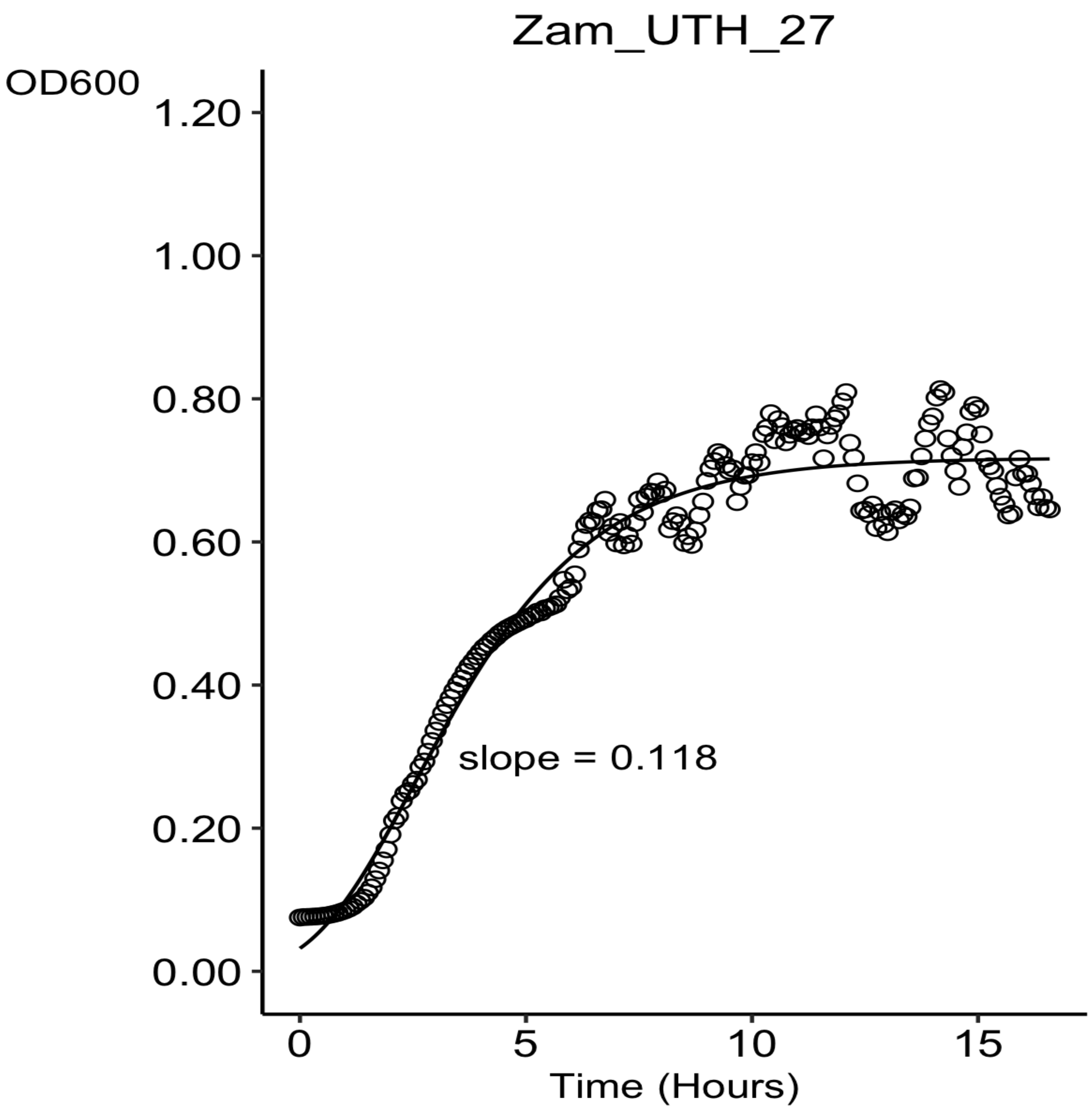
Zam_UTH_27
OD600
1.20
1.00
0.80
0.60
0.40
0.20
0.00
slope = 0.118
0
5
10
15
Time (Hours)

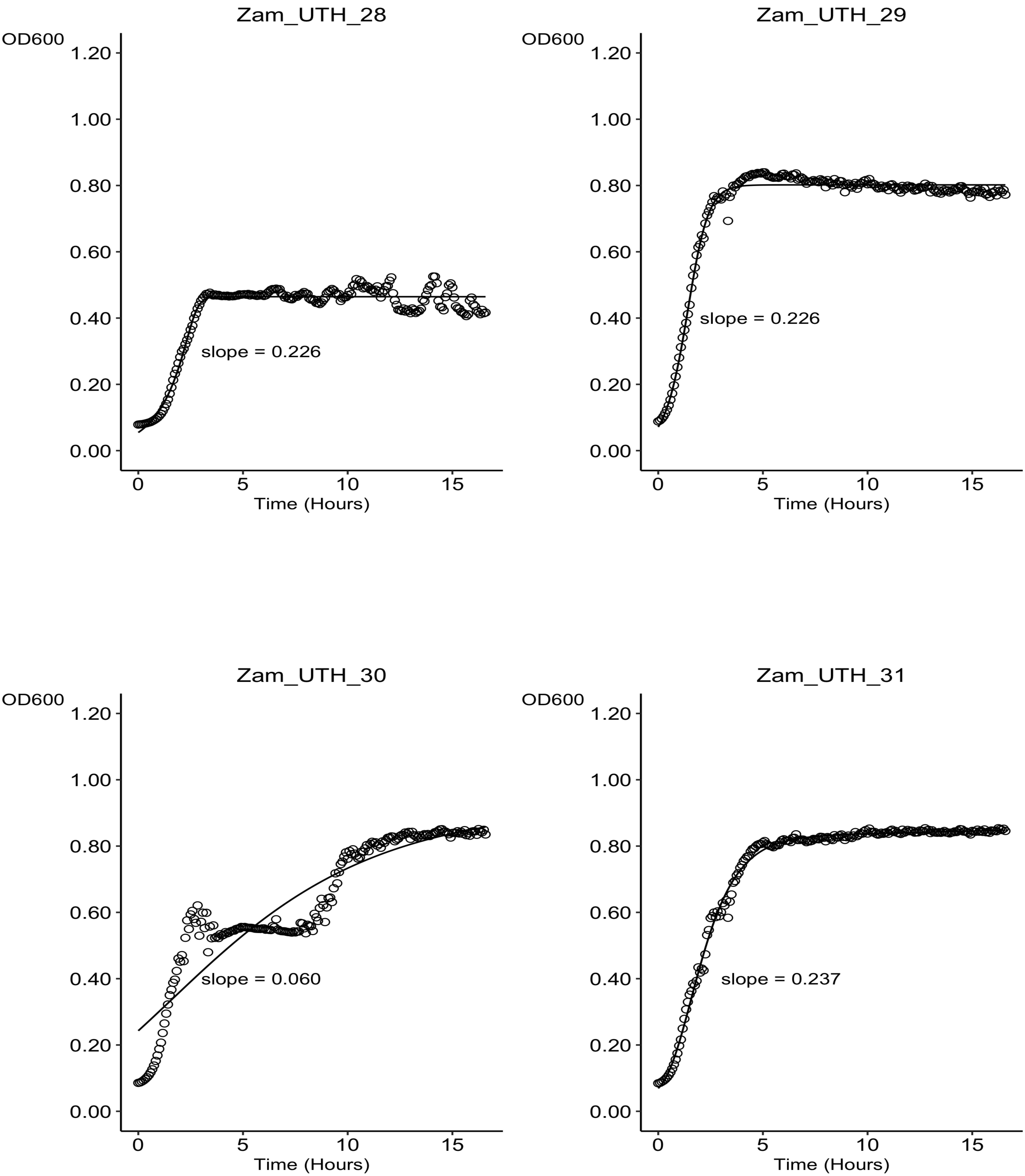
Zam_UTH_28
OD600
1.20
1.00
0.80
0.60
0.40
0.20
0.00
slope = 0.226
0
5
10
15
Time (Hours)
Zam_UTH_29
OD600
1.20
1.00
0.80
0.60
0.40
0.20
0.00
slope = 0.226
0
5
10
15
Time (Hours)
Zam_UTH_30
OD600
1.20
1.00
0.80
0.60
0.40
0.20
0.00
slope = 0.060
0
5
10
15
Time (Hours)
Zam_UTH_31
OD600
1.20
1.00
0.80
0.60
0.40
0.20
0.00
slope = 0.237
0
5
10
15
Time (Hours)

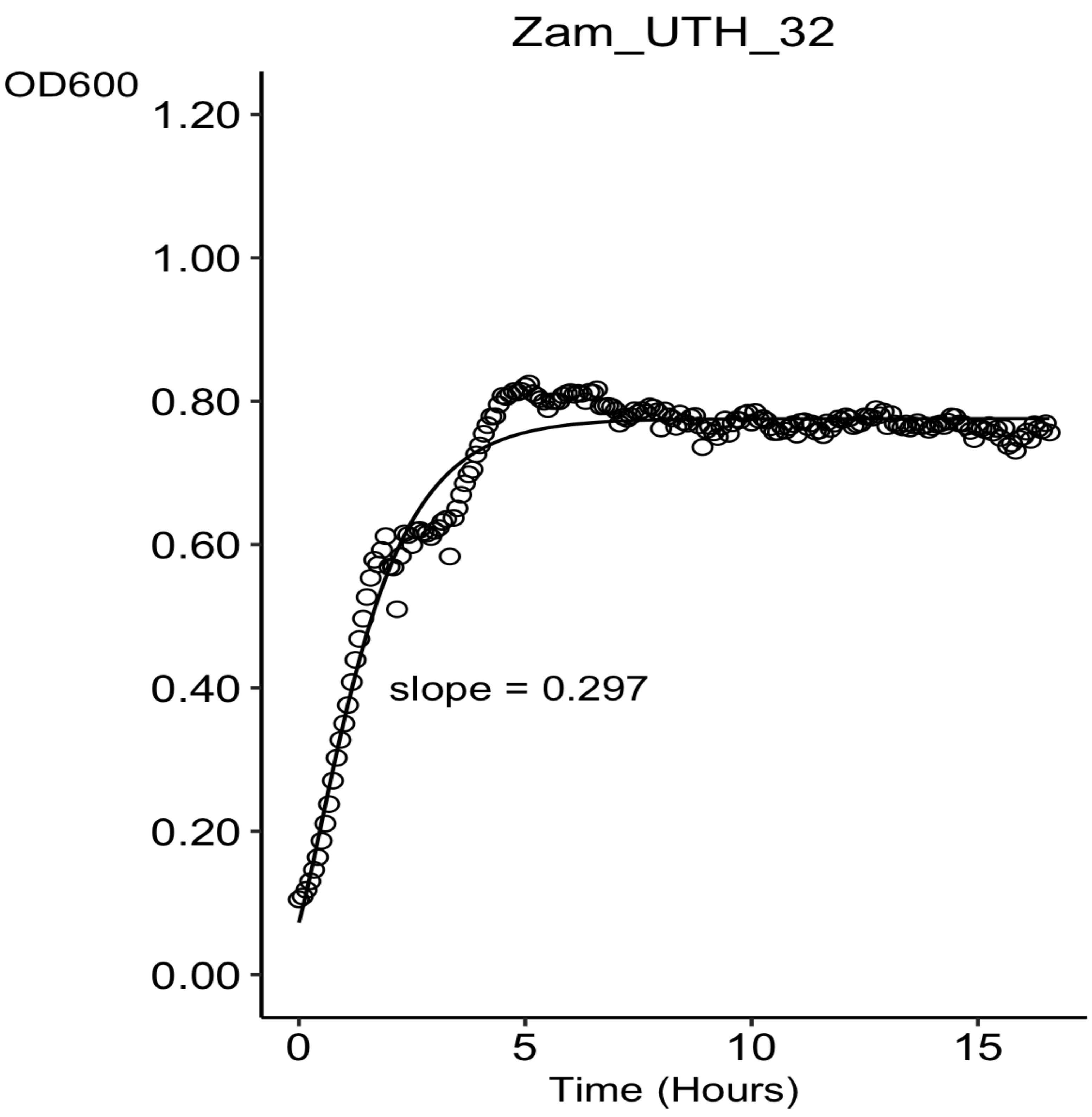
Zam_UTH_32
OD600
1.20
1.00
0.80
0.60
0.40
0.20
0.00
slope = 0.297
0
5
10
15
Time (Hours)

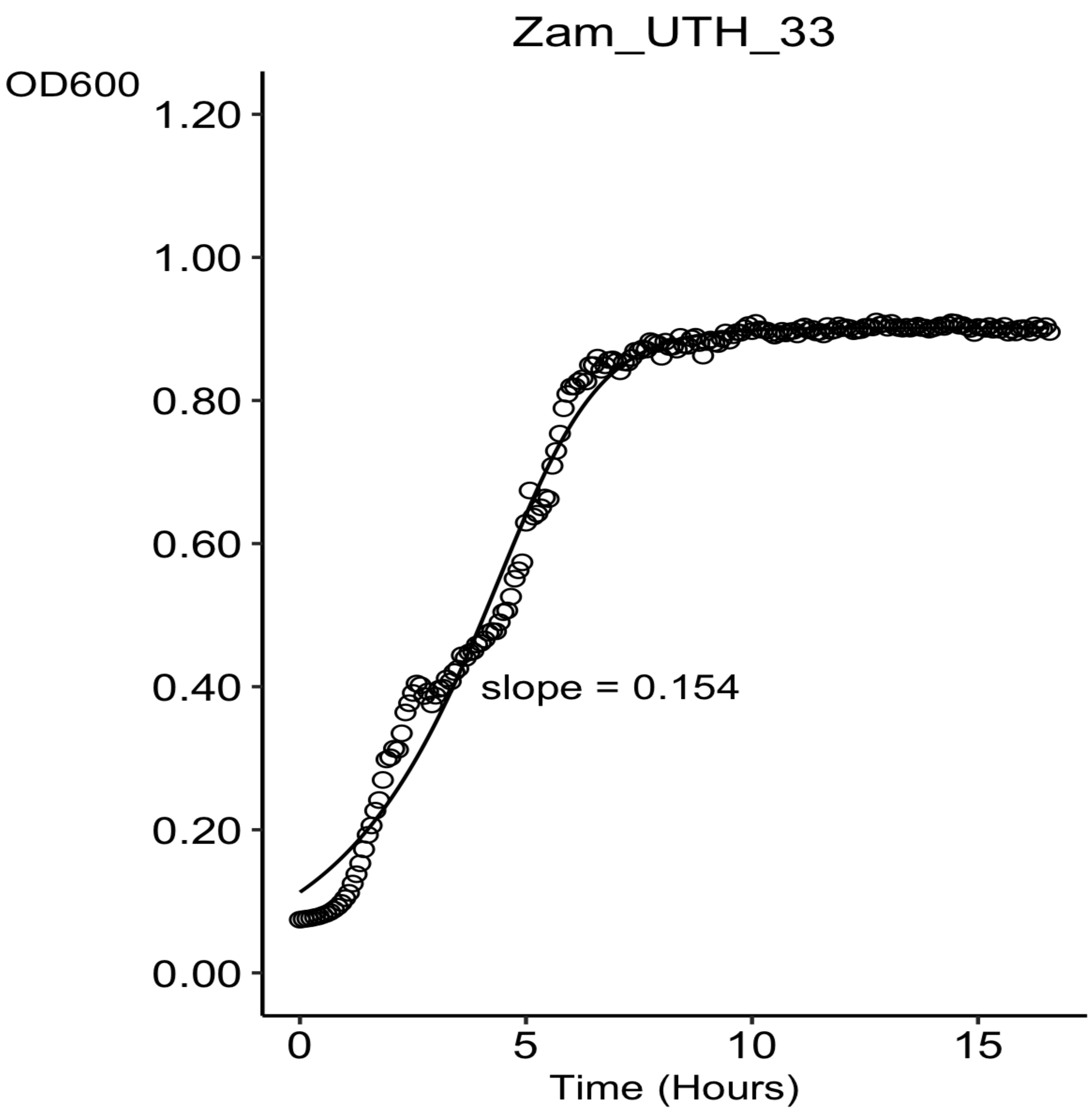
Zam_UTH_33
OD600
1.20
1.00
0.80
0.60
0.40
0.20
0.00
slope = 0.154
0
5
10
15
Time (Hours)

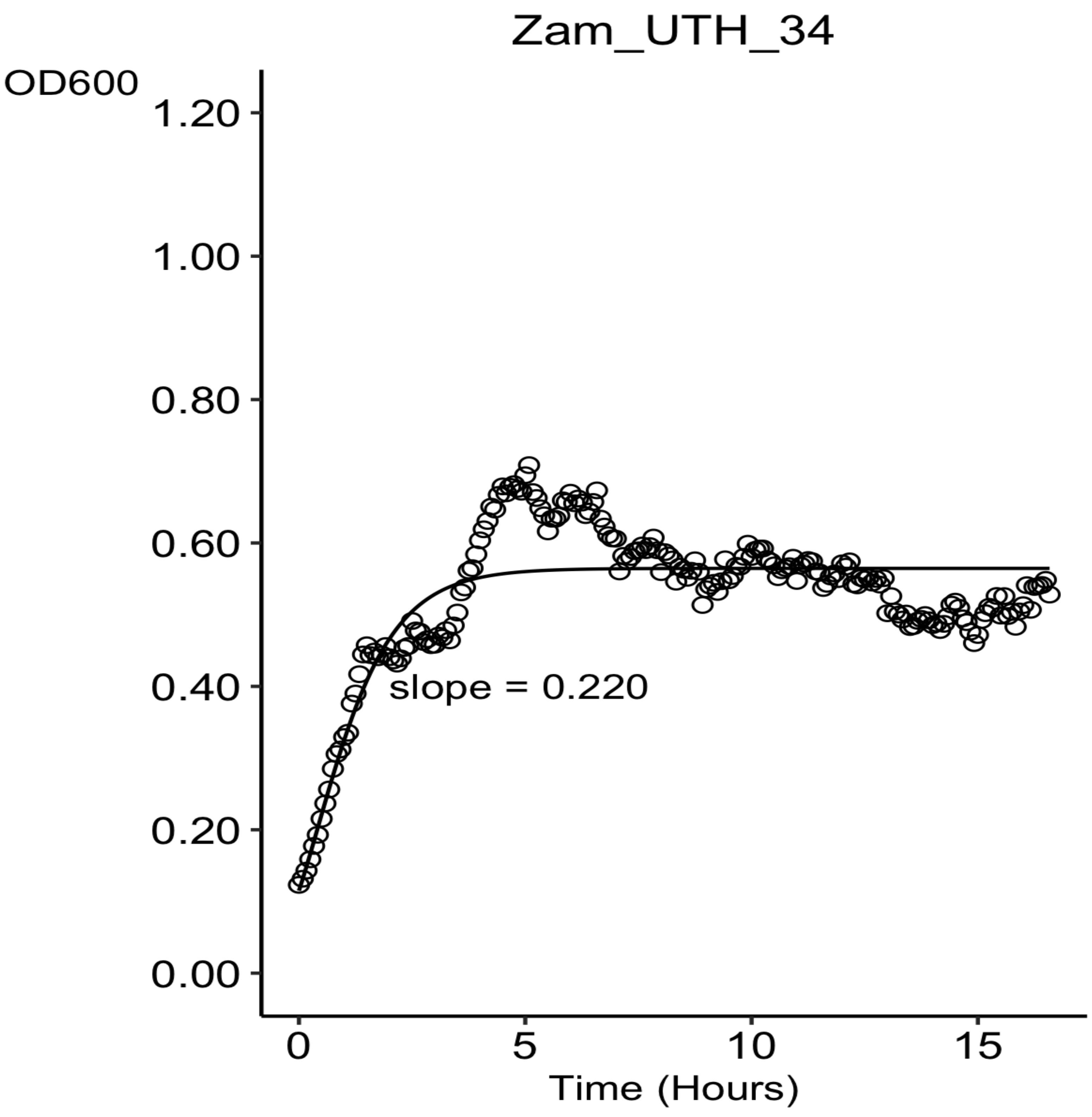
Zam_UTH_34
OD600
1.20
1.00
0.80
0.60
0.40
0.20
0.00
slope = 0.220
0
5
10
15
Time (Hours)

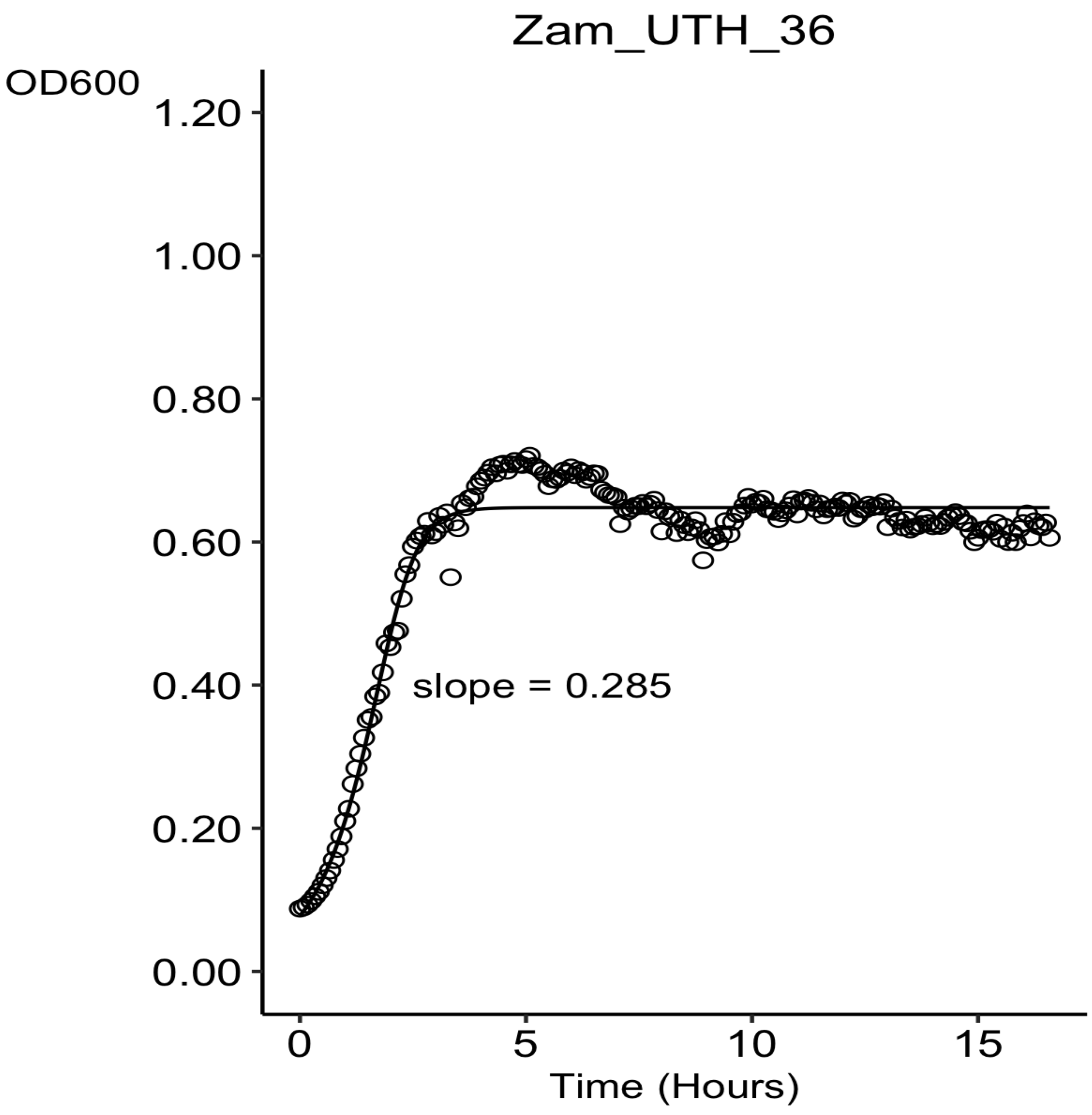
Zam_UTH_36
OD600
1.20
1.00
0.80
0.60
0.40
0.20
0.00
slope = 0.285
0
5
10
15
Time (Hours)

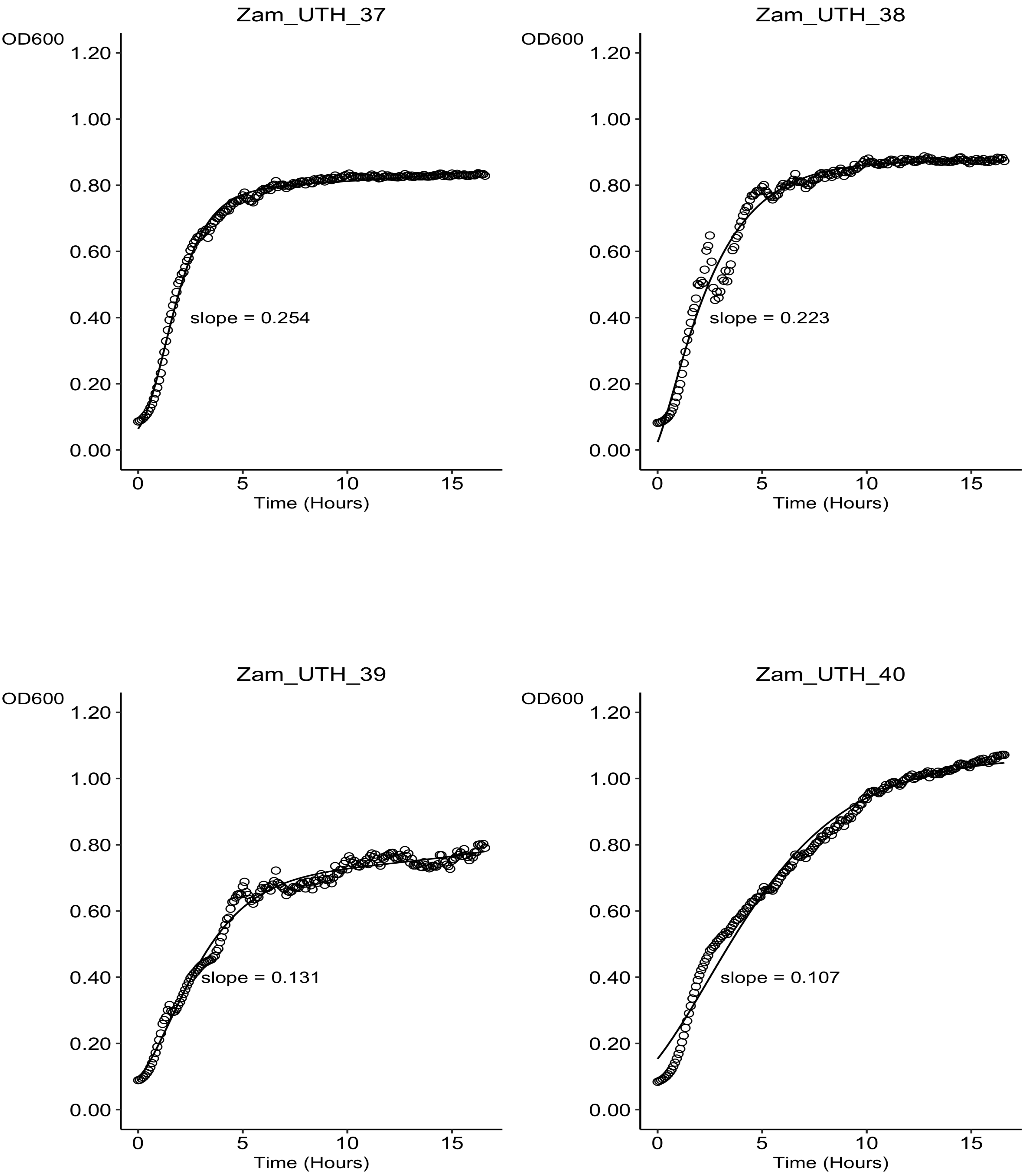
Zam_UTH_37
OD600
1.20
1.00
0.80
0.60
0.40
0.20
0.00
slope = 0.254
0
5
10
15
Time (Hours)
Zam_UTH_38
OD600
slope = 0.223
Time (Hours)
Zam_UTH_39
OD600
slope = 0.131
Time (Hours)
Zam_UTH_40
OD600
slope = 0.107
Time (Hours)

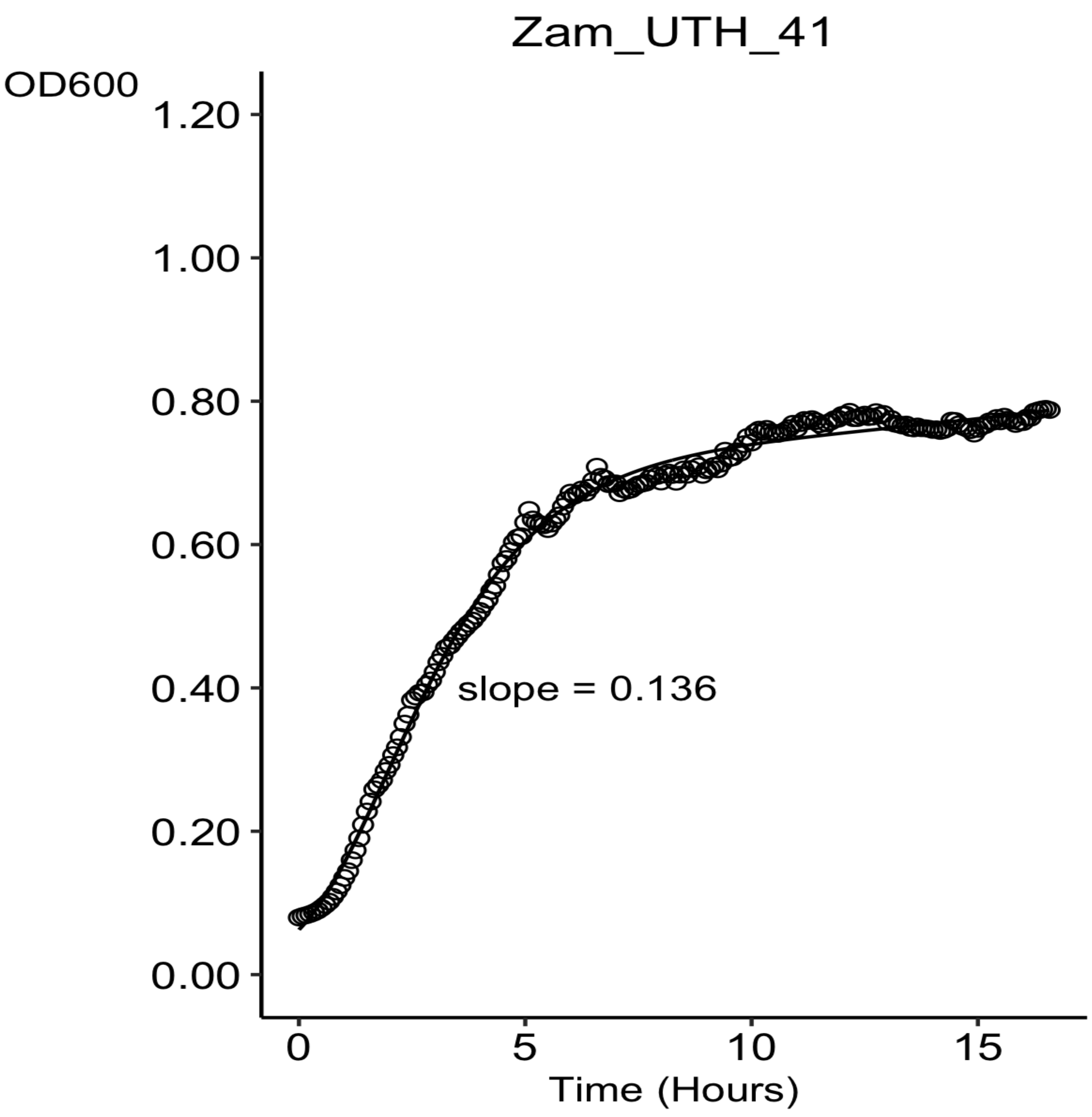
Zam_UTH_41
OD600
1.20
1.00
0.80
0.60
0.40
0.20
0.00
slope = 0.136
0
5
10
15
Time (Hours)

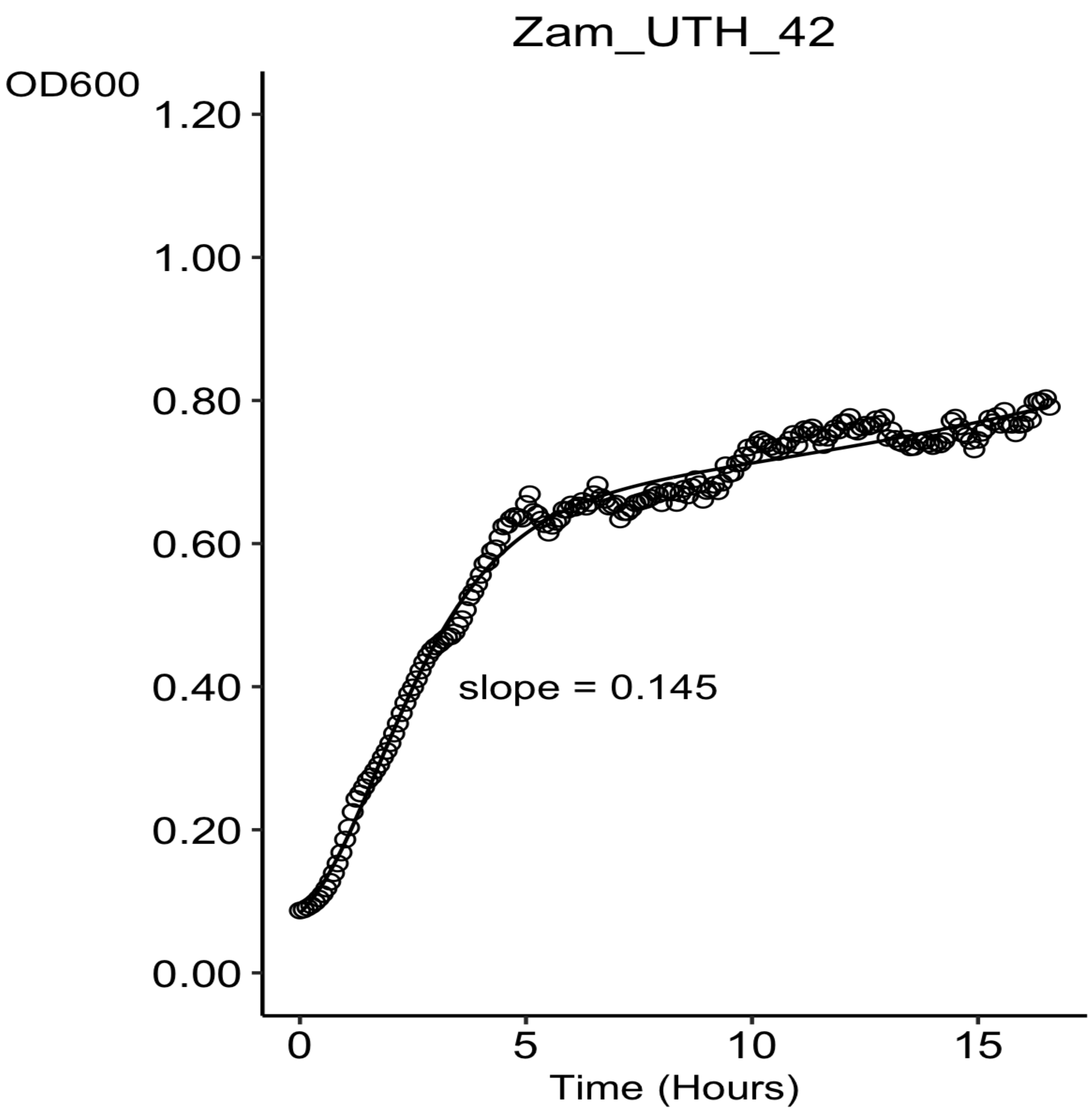
Zam_UTH_42
OD600
1.20
1.00
0.80
0.60
0.40
0.20
0.00
slope = 0.145
0
5
10
15
Time (Hours)

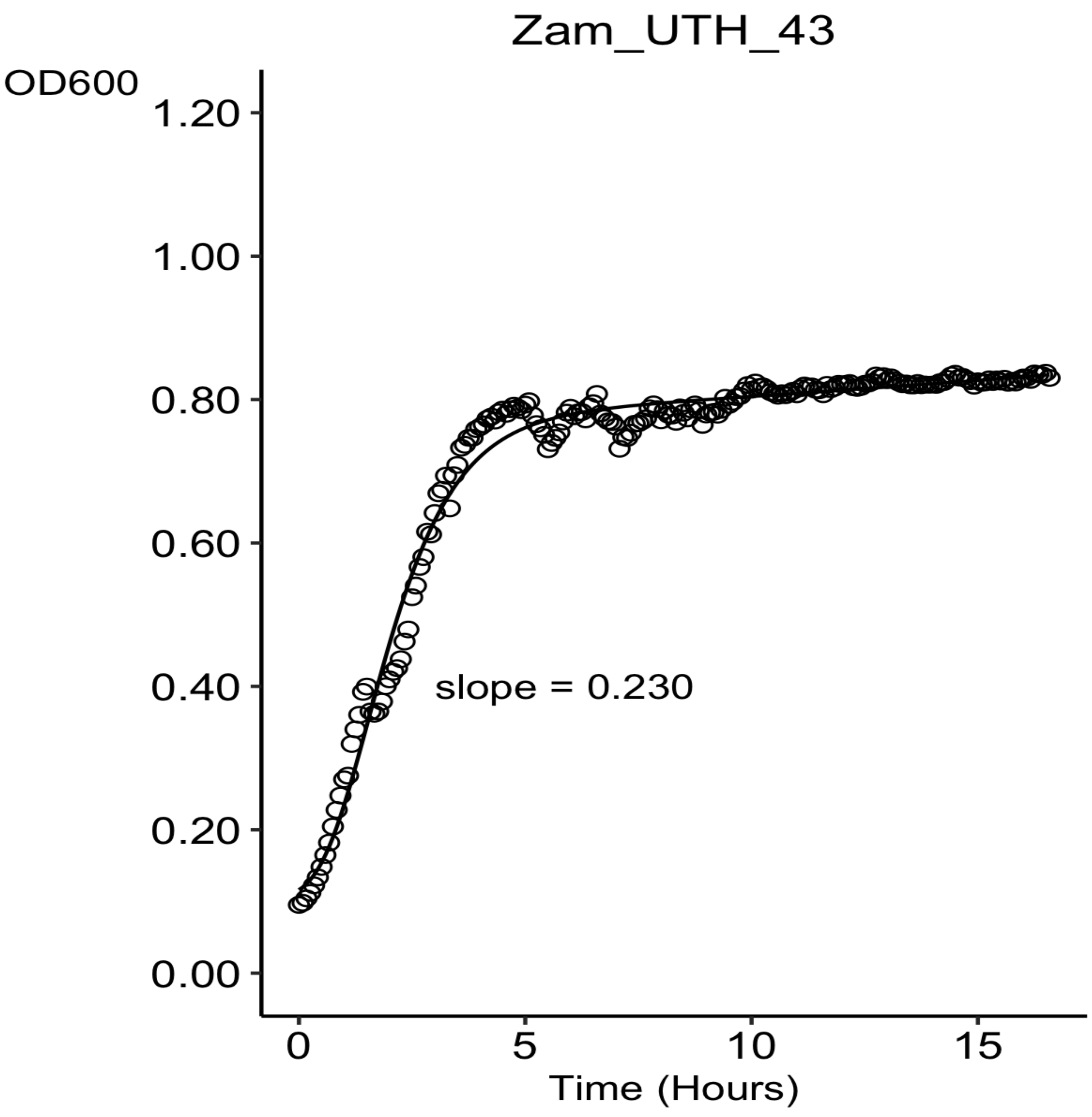
Zam_UTH_43
OD600
1.20
1.00
0.80
0.60
0.40
0.20
0.00
slope = 0.230
0
5
10
15
Time (Hours)

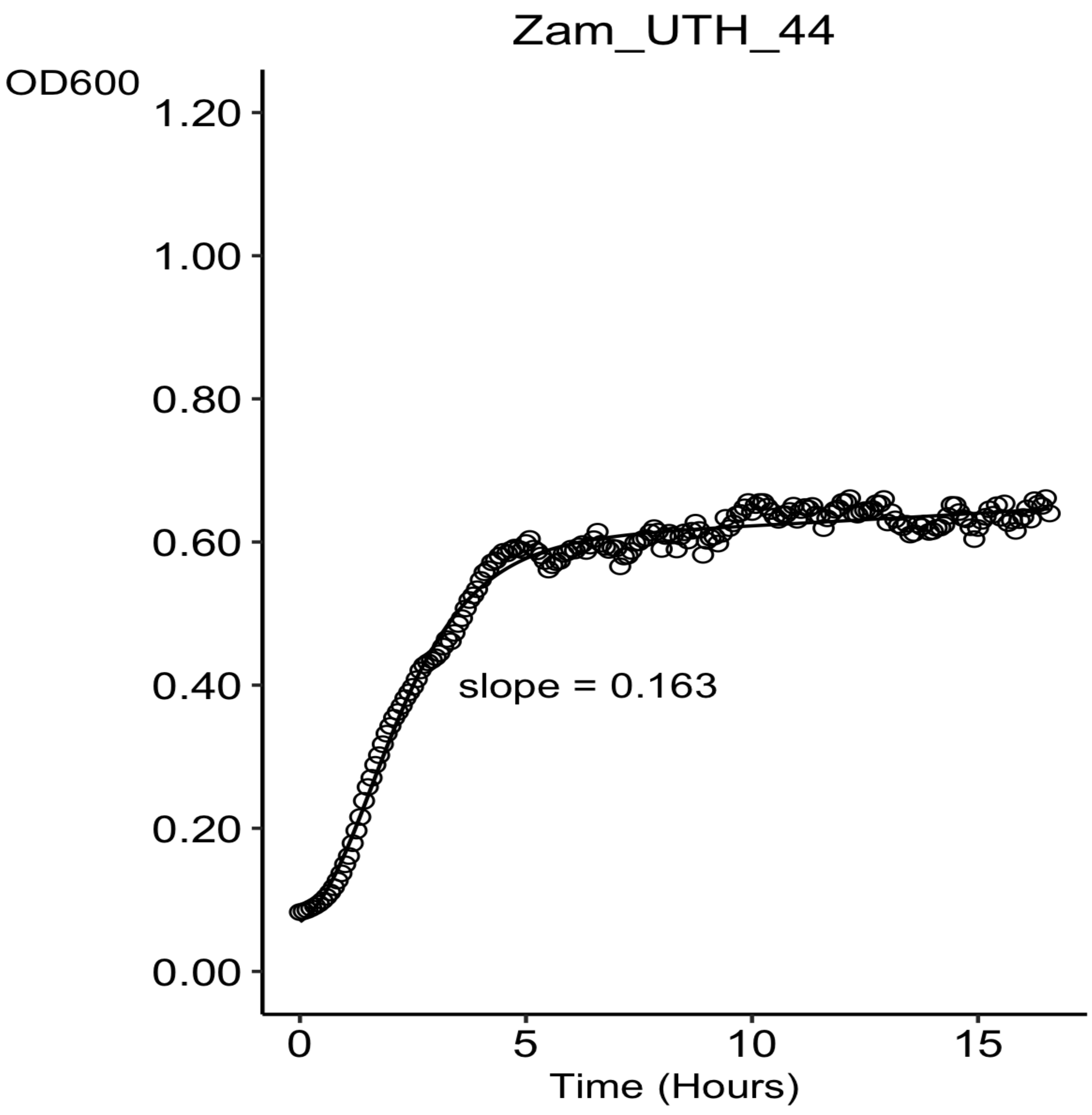
Zam_UTH_44
OD600
1.20
1.00
0.80
0.60
0.40
0.20
0.00
slope = 0.163
0
5
10
15
Time (Hours)

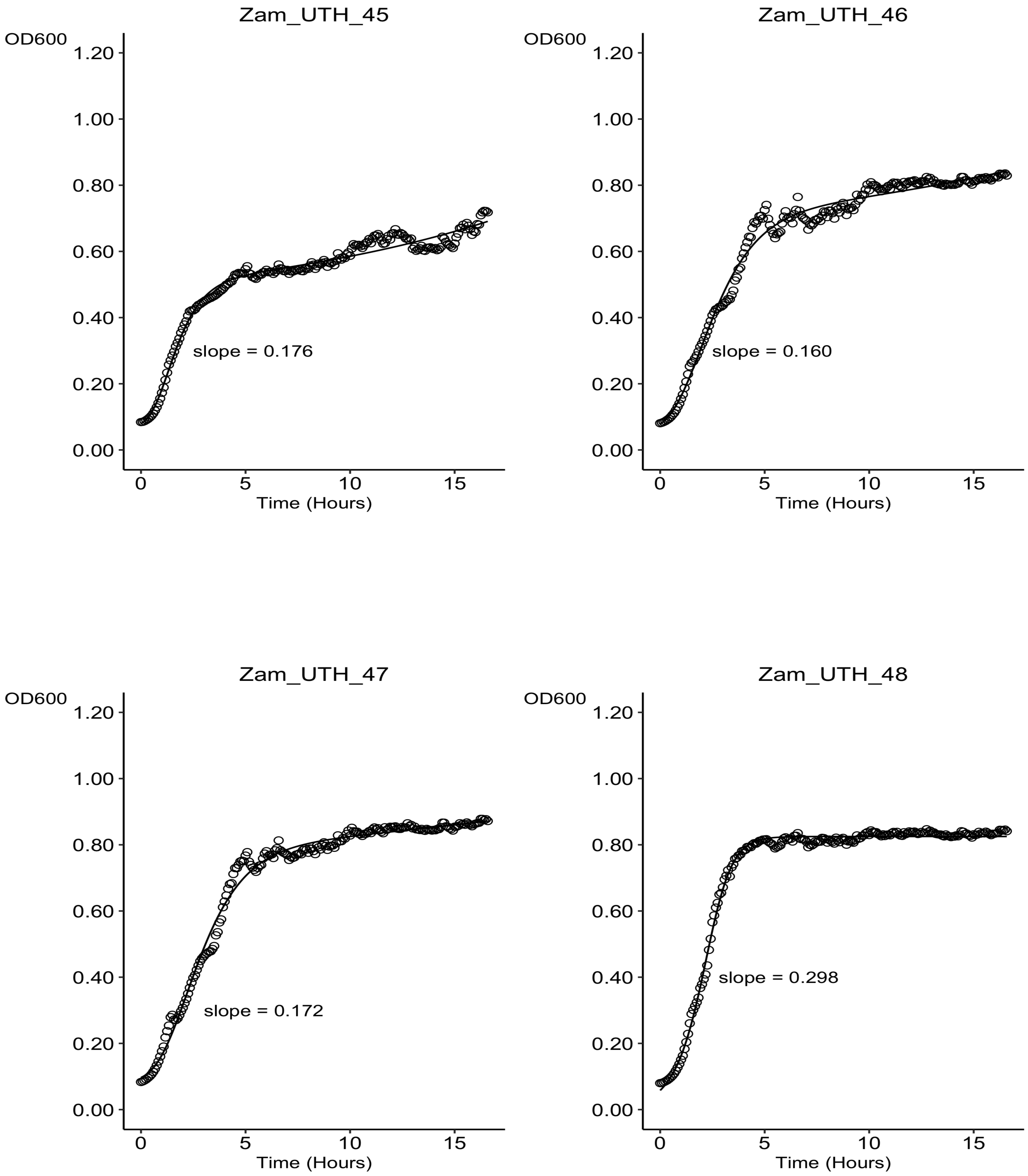
Zam_UTH_45
OD600
1.20
1.00
0.80
0.60
0.40
0.20
0.00
slope = 0.176
0
5
10
15
Time (Hours)
Zam_UTH_46
OD600
1.20
1.00
0.80
0.60
0.40
0.20
0.00
slope = 0.160
0
5
10
15
Time (Hours)
Zam_UTH_47
OD600
1.20
1.00
0.80
0.60
0.40
0.20
0.00
slope = 0.172
0
5
10
15
Time (Hours)
Zam_UTH_48
OD600
1.20
1.00
0.80
0.60
0.40
0.20
0.00
slope = 0.298
0
5
10
15
Time (Hours)

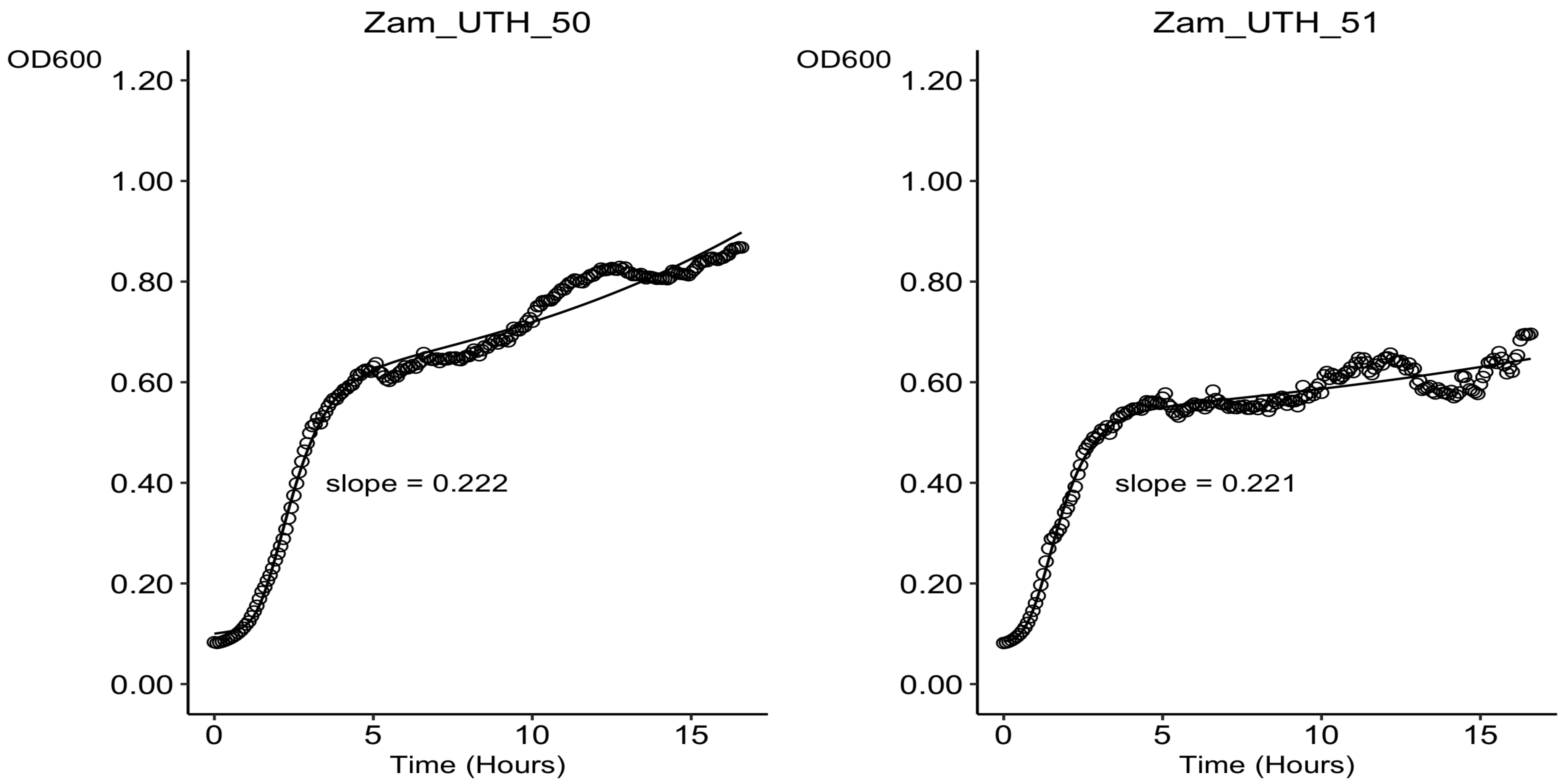

Supplementary Figure 5. Growth rates for 46 strains used in this study
Growth rates were estimated from slopes obtained by fitting growth curves from optical density data at different times.
